# Identifying Risk and Protective Factors Impacting the Clinical Outcomes of Subthreshold Anxiety in Early Adolescents: Insights From the ABCD Study

**DOI:** 10.1155/da/6514030

**Published:** 2025-06-25

**Authors:** Chen Keyin, Li Qian, Zhang Jiayuan, Niu Lijing, Dai Haowei, Peng lanxin, Wang Xingqin, Ma Qing, Zhang Ruibin

**Affiliations:** ^1^Laboratory of Cognitive Control and Brain Healthy, Department of Psychology, School of Public Health, Southern Medical University, Guangzhou, China; ^2^Department of Neurosurgery, Institute of Brain Diseases, Nanfang Hospital of Southern Medical University, Guangzhou, China; ^3^School of Psychology and Cognitive Science, East China Normal University, Shanghai, China; ^4^Guangdong-Hong Kong-Macao Greater Bay Area Center for Brain Science and Brain-Inspired Intelligence, Guangdong-Hong Kong Joint Laboratory for Psychiatric Disorders, Guangdong Basic Research Center of Excellence for Integrated Traditional and Western Medicine for Qingzhi Diseases, Guangzhou, China; ^5^Department of Psychiatry, Zhujiang Hospital, Southern Medical University, Guangzhou, China

**Keywords:** adolescent brain and cognitive development study, anxiety disorder, prevention, prognosis, remission, subthreshold anxiety

## Abstract

**Background:** Subthreshold anxiety (STA) is a significant risk factor for developing anxiety disorder (AX), particularly in adolescence. Understanding the risk and protective factors of the development of STA in early life is essential for early prevention and intervention efforts. However, research on this topic is scarce.

**Methods:** We examined the data of 11,876 early adolescents from the Adolescent Brain and Cognitive Development (ABCD) Study to explore the factors influencing the development of STA between ages 9 and 13. The outcomes included developing AX, persistent STA, and remission from STA. Using the Child Behavior Checklist (CBCL), we identified 786 participants with STA. To predict STA transitions, we analyzed 31 diathesis-stress-related variables covering demographics, mental and physical health, and environmental factors, employing logistic regression.

**Results:** Compared to baseline healthy controls (HCs), adolescents with STA showed an odds ratio (OR) of 6.9 for converting to AX. The pivotal risk factors for progression from STA to AX were lack of perseverance and area deprivation, with females being more likely to maintain STA. Protective factors for a favorable prognosis of STA included the absence of traumatic history, lack of premeditation, increased physical activity, and positive school environment.

**Conclusions:** Healing traumatic experiences, increased physical activity, and enhancing school and family environments could help prevent adverse outcomes. By targeting these modifiable factors, adolescents at high risk can be identified and provided with interventions early in life.

## 1. Introduction

Anxiety disorders (AXs) involve excessive fear and anxiety and related behavioral disturbances, with symptoms that are severe enough to result in significant distress or significant impairment in personal, family, social, educational, occupational, or other important areas of functioning, as defined by the ICD-11 diagnostic criteria [1]. AX is highly prevalent in children and adolescents, with a 7% prevalence rate in epidemiological studies [2]. In addition to its high prevalence, AX has the earliest age of onset compared to other major mental health disorders and, if left untreated, can persist into adulthood, resulting in significant personal and societal costs [3].

According to transdiagnostic model, AX exists in a continuum of increasingly severity [4]. Transdiagnostic dimensional approaches apply continuous (versus categorical or dichotomous) dimensions to psychopathology data, which represent unbroken spectra (also referred to as factors) that range from very low to very high levels (and all levels in between) [5]. A clinical staging model was tailored to AX, distinguishing two main stages of disease progression varying from subclinical stages to clinical stages [6]. When viewed as a spectrum, an individual who presents with clinically relevant symptoms of anxiety but does not meet the diagnostic criteria for threshold anxiety can be diagnosed with subthreshold anxiety (STA) [7]. The prevalence of STA ranges from 8.5% to 11.4% [8], and it significantly impairs mental and physical functioning in individuals, leading to an increased need for medical care among those with STA. In one study, a large proportion of the patients experienced persistent symptoms or progressed to full-blown AX at follow-up [9, 10]. Therefore, not only risk factors for disorder onset but also predictors for unfavorable course trajectories of anxiety need to be examined to identify high-risk individuals who might benefit from targeted early interventions at initial stages [11].

The different outcomes of STA include “transition to AX,” “persistence of STA,” and “remission from STA” [12, 13]. Identifying specific factors that contribute to these outcomes is crucial for informing targeted interventions. Protective factors, such as strong social support systems and early therapeutic interventions, can mitigate the risk of adverse outcomes, while risk factors like chronic stress and comorbid mental health conditions may increase the likelihood of persistence of STA or transition to AX [11, 14].

Early adolescence is the core risk phase for the development of anxiety symptoms and syndromes, ranging from transient mild symptoms to full-blown AX [15]. Subthreshold forms of AX (e.g., fearful spells) often manifest considerably earlier in childhood or early adolescence [16]. Assessing the prevalence of STA during this developmental stage provides a baseline for understanding its burden, while examining its course allows to identify patterns of persistence, remission, or progression to AX over time. Identifying risk factors or enhancing protective factors in early adolescents represents the most promising window for preventing AX, given the potential for behavioral patterns established during this period to persist over the life course and influence long-term mental health trajectories [17]. Therefore, by assessing the prevalence and course of STA, as well as exploring predictive factors associated with its development during this critical period, we aimed to precisely identify individuals with STA who are at increased risk for adverse long-term outcomes.

The etiology of anxiety involves a multifactorial interaction between genetic vulnerability and environmental factors, such as stressful situations. Therefore, we employed the diathesis-stress model to address this issue [18]. The diathesis-stress model posits an interaction between stressors and personality traits, where personality vulnerability increases the likelihood of psychopathology only when combined with significant stressors [19]. Diatheses (stable vulnerability factors like genetic predisposition or temperament) create latent risk, and stressors (environmental triggers like academic pressure or social evaluation) activate this vulnerability. Their interaction determines symptom onset and trajectory. This model can provide a comprehensive framework for understanding the complex interplay between individual characteristics and environmental factors in the development of anxiety symptoms.

In this study, we conducted an investigation of a wide range of diathesis-stress-related variables in a large sample (*N* = 11,876) of early adolescents (9–11 years old) from the US-based Adolescent Brain and Cognitive Development (ABCD) Study [20]. We employed three patterns (STA → AX vs. healthy controls (HCs) to identify the predictors of progression to AX; STA → STA vs. HCs to identify the predictors of persistent STA; STA → HCs vs. STA → AX to identify the predictors of remission from STA) to discern the predictors of the different outcomes of STA. Previous studies [14, 21] have identified relevant risk and protective factors related to STA in adults. Predictors of incident AX have been extensively studied, and several individual, familial, and environmental variables were identified as risk factors for anxiety [22]. However, anxious children and adolescents may also experience unique psychosocial risks, and there is a dearth of information on the risk and protective factors for the different transformations of STA in the early adolescent population. Therefore, the aims of this study were twofold. First, we assessed the prevalence, characteristics, and 3-year course of STA in early adolescents using data from the ABCD study. We predicted that anxiety symptoms would largely exhibit continuity over the course of the study, and we sought to investigate the continuity and change of STA. Second, we explored the risk and protective factors for the different development of early adolescents STA, including demographic and diathesis- and stress-related variables. This approach, grounded in the diathesis-stress model, allowed us to identify not only the inherent vulnerabilities that predispose individuals to anxiety but also the specific stressors that may trigger or exacerbate these symptoms. By providing a more nuanced understanding of how these factors interact, our study aimed to inform targeted prevention strategies and mechanistic research on anxiety persistence and progression, ultimately contributing to more personalized and effective interventions for adolescent anxiety.

## 2. Methods

### 2.1. Sampling

Data were collected from the ABCD study (release 5.0) in the United States, which is an ongoing, nationwide study of the effects of demographics, physical health, mental health, and environmental influences on behavioral and brain development in adolescents (https://data-archive.nimh.nih.gov/abcd).

At the baseline between 2016 and 2018, a representative sample of *N* = 11,876 individuals was interviewed. Subsequent follow-up assessments were conducted annually. In the current study, data collected at the baseline (ages 9–10), 1-year follow-up (ages 10–11), 2-year follow-up (ages 11–12), and 3-year follow-up (ages 12–13) were utilized to form a study sample consisting of *N* = 786 eligible participants. The data of predictor variables were collected from baseline.

**Figure 1:**
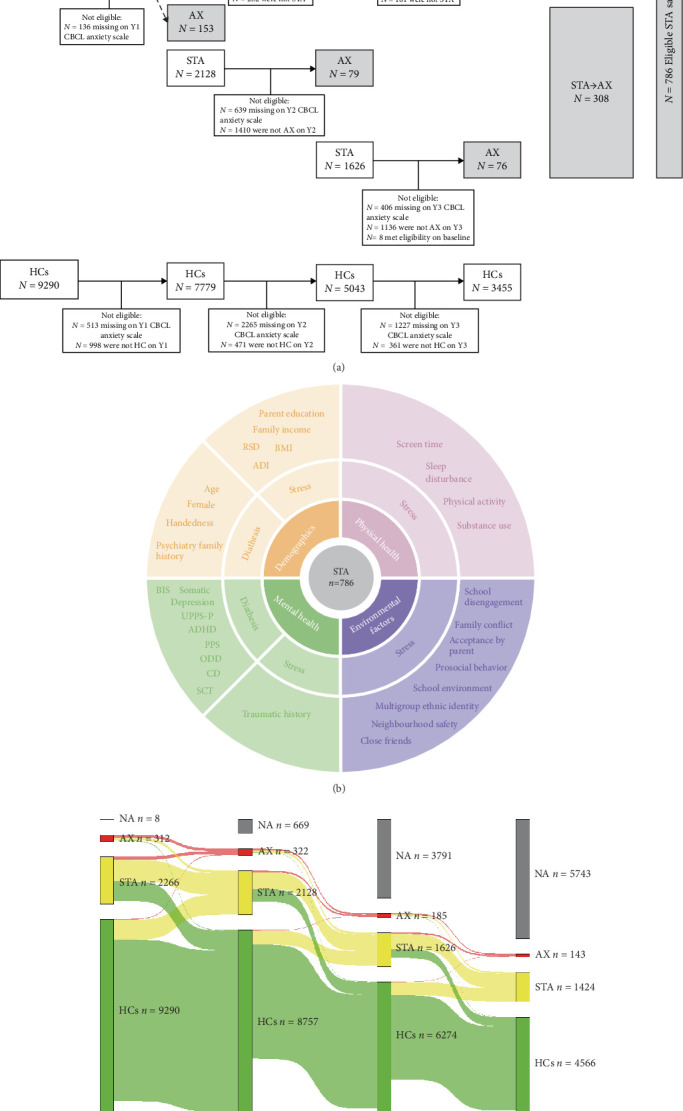
Sampling, longitudinal outcome, and predictors of subthreshold anxiety in the Adolescent Brain and Cognitive Development Study. (A) Sampling subthreshold anxiety in the Adolescent Brain and Cognitive Development Study. Among the 786 eligible participants with subthreshold anxiety (highlighted in gray), the STA → AX group showed an increase in numbers over each wave, while all other groups displayed a decrease in ineligible subjects over time. The STA → HCs group needed to fulfill the criteria of having subthreshold anxiety at baseline, transitioning to a healthy control (HC) status in the subsequent year, and maintaining this status for the following 2 years. The STA → STA group was required to maintain subthreshold anxiety from baseline to the subsequent 3 years, and the HC group had to maintain HC status over the same 3-year period. (B) Predictors from demographics, physical health, mental health, and environment variables. (C) Natural course and longitudinal outcome of anxiety symptoms change over 3 years among 11,876 adolescents illustrated through sankey diagram. NA presents the missing data. ADI, area deprivation index; AX, anxiety disorder; BIS, behavioral inhibition; BL, baseline; CD, conduct disorder; HCs, healthy controls; NA, not available; ODD, oppositional defiance disorder; PPS, prodromal psychosis scale; RSD, recent social deprivation; SCT, sluggish cognitive tempo; STA, subthreshold anxiety; UPPS-LP, lack of premeditation of UPPS-P impulsive behavior scale; Y1, year 1; Y2, year 2; Y3, year 3.

Participant selection criteria in this study were as follows. At the baseline (*N* = 2266), 1-year follow-up (*N* = 2128), and 2-year follow-up (*N* = 1626), individuals with STA who progressed to AX in the subsequent year were categorized into the STA→AX group (*N* = 308). Among baseline individuals with STA (*N* = 2266), those who maintained STA during the 3-year follow-up period were included in the STA→STA group (*N* = 229), and those who transitioned to HC status at the 1-year follow-up and maintained this state in the following years were classified into the STA→HCs group (*N* = 249). Among baseline HCs (*N* = 9290), those who maintained a healthy status during 3-year follow-up period were included in the HCs group (*N* = 3455). The exclusion criteria in the participant screening process were individuals who did not meet the corresponding status at follow-up and those with missing scale data at follow-up. The inclusion criterion was individuals who met the category defined by the CBCL-AP scale threshold at follow-up. The flowchart illustrating the inclusion of participants can be found in Figure 1A. Additionally, differences in variables across the four groups (STA → AX, STA → STA, STA → HCs, and HCs), as well as between eligible STA participants and excluded STA groups, are detailed in Tables S1 and S2.

### 2.2. Measures

#### 2.2.1. Prognosis of STA

The prognosis of STA at baseline was assessed as per three outcome variables using the CBCL (parents report) [23]. The outcome variables included “transition to AX,” “persistence of STA,” and “remission from STA.” The CBCL is a standardized instrument that has been widely used to identify participants exhibiting anxiety symptoms. It serves multiple purposes, including aiding in the assessment of symptom severity, functioning as a screening measure for potential anxiety, and providing essential data for characterizing the patterns of anxiety symptoms [24]. Notably, our classification relied exclusively on the CBCL, whose behavioral problem scales are uniquely suited to capture childhood anxiety manifestations (e.g., avoidance) and prodromal markers [25]. Its continuous scaling system enabled granular severity quantification—critical for detecting STA—unlike categorical measures (e.g., KSADS). Combined with robust psychometrics (test–retest reliability = 0.94; validity = 0.92; [25]) and superior ABCD data completeness, the CBCL was ideal for analytic rigor.

#### 2.2.2. STA

The participants were deemed to have STA when the t-scores of the CBCL–anxiety scale were 55–69 [26].

#### 2.2.3. AX

Participants with a *t*-score of 70 or higher were classified as having an AX [26].

#### 2.2.4. HCs

HCs refer to participants with t-score on the CBCL–anxiety scale lower than 55 [26]. Remission from STA refers to returning to a healthy state.

#### 2.2.5. Predictors

In total, 31 putative predictors were selected, encompassing various domains such as demographics (age, sex, handedness, family income, psychiatric family histories, parent education, area deprivation index [ADI], recent social deprivation [RSD], and body mass index [BMI]), physical health (screen time, physical activity, sleep disturbances, and substance use), mental health (behavioral inhibition, UPPS-P impulsive behavior, prodromal psychosis, depression, somatic problems, and attention-deficit/hyperactivity disorder [ADHD], oppositional defiance, conduct disorder, sluggish cognitive tempo, and traumatic experiences), and environmental factors (parents' multigroup ethnic identity, neighborhood safety, family conflict, prosocial behavior, acceptance by parents, school environment, school disengagement, and close friends) (Tables S3 and S4). The predictor selection was theoretically anchored in the diathesis-stress framework [18], with specific operationalizations informed by its integration with cognitive–behavioral theory: Maladaptive cognitive schemas (diatheses) were selected based on Beck and Weishaar's [27] formulation of how enduring negative beliefs interact with stressful events to produce anxiety symptoms, particularly through distorted threat appraisal processes.

#### 2.2.6. Statistical Analysis

Initially, we computed the occurrence rates of AX by categorizing respondents based on the presence or absence of STA at baseline as well as a history of AX. Subsequently, we determined the odds ratios (ORs) using the calcOddsRatio function to evaluate how subthreshold symptoms and past history influenced the development of AX during the 3-year follow-up period. Missing data were imputed using the mice function in R version 4.2.1, which executed the multiple imputation process and returned multiple complete datasets automatically.

We utilized multinomial logistic regression to model the predictors for all categories: STA → AX, STA → STA, STA → HCs, and HCs, simultaneously, providing a comprehensive view of the predictors across these groups (Table S5). Then, we employed logistic regression analysis (using the glm function) to quantify statistically significant associations among participants' demographics, physical health, mental health, and environment characteristics. In our analysis, we included “site” as a covariate to control for potential site-related variability and ensured that our findings were not due to unmeasured differences between sites. Initially, we identified factors at baseline that distinguish individuals with STA from HCs. Subsequently, we removed these shared variables in the subsequent logistic regression analysis to explore specific factors that predict differences in trajectories over time for those with STA at ages 9–10 years. To measure the significance difference between variables, we utilized three patterns: STA → AX vs HCs; STA → STA vs HCs; STA → HCs vs STA → AX [22]. In the STA → AX vs HCs pattern, we designated participants who developed AX at follow-up as the “positive case” for predicting AX from STA. Conversely, those who consistently remained in HC status were considered the “negative case”. In the STA → STA vs HCs pattern, we identified participants who persisted with chronic STA at follow-up as the “case group” for predicting the continuation of chronic STA. Participants who consistently remained in HC status were classified as the “control group”. In the STA → HCs vs STA → AX pattern, participants who remitted from STA at follow-up were identified as the “negative case” for predicting remission from STA. Participants who developed AX were classified as the “positive case”. Continuous variables were standardized to generate *z*-scores with a mean of 0 and a standard deviation (SD) of 1. Collinear variables with correlations exceeding 0.5 were excluded [28]. A bootstrapping approach was employed to ensure generalizability across sites, address the unequal sample sizes in the groups, and correct for multiple comparisons [29]. Bootstrapping is an effective technique to handle complex datasets without relying on specific assumptions or prior knowledge [30]. Each regression analysis was bootstrapped 10,000 times. For every iteration, we extracted the *β* values, Wald statistics, *p*-values, ORs, and corresponding 95% confidence intervals (CIs) for each variable. Subsequent logistic regression analyses were conducted on significant variables (*p* < 0.05) to calculate the OR for each significant variable, elucidating the relationship between the variable and the dependent variable. The statistical significance of a variable was determined by the number of iterations reporting *p* < 0.05 for that variable, thereby correcting for multiple comparisons across the 10,000 random subsamples. Variables were considered significant at *p* < 0.05 following Bonferroni correction for multiple testing.

### 2.3. Concordance Analysis

After using the CBCL to diagnose and determine predictive factors of STA's transition, we then utilized a different diagnostic method, the KSADS-5, to confirm the diagnosis and conduct the same logistic analysis (for detailed information, see supporting information). The KSADS-5 is administered via computer in ABCD study, which is effective for assessing the anxiety states of adolescents. Test–retest reliability kappa coefficients were in the excellent range for present and/or lifetime diagnoses of major depression, any bipolar, generalized anxiety, conduct, and oppositional defiant disorder (0.77–1.00), suggested that the KSADS generates reliable and valid child psychiatric diagnoses [31]. The inter-rater reliability kappa value of the scale was above 0.8 [32]. Comparing the predictors obtained from both diagnostic methods (CBCL and KSADS-5; kappa = 0.06), we aimed to identify similar factors to enhance reliability of the results.

## 3. Results

### 3.1. Prognosis of STA

At baseline, 2266 participants experienced STA. In the 1-year follow-up, 1035 (45.7%) participants remained in STA, 942 (41.6%) transitioned to HCs, and 153 (6.8%) transitioned to AX; 2128 participants had STA in the 1-year follow-up. In the 2-year follow-up, 797 (37.5%) participants remained in STA, 613 (28.8%) transitioned to HCs, and 79 (3.7%) transitioned to AX; 1626 participants had STA in the 2-year follow-up. In the 3-year follow-up, 705 (43.4%) participants remained in STA, 439 (27.0%) transitioned to HCs, and 76 (4.7%) transitioned to AX. Overall, among those with STA, the largest proportion maintained chronic STA, a significant number experienced remission, and a small proportion developed AX (Figure 1C).

Adolescents with STA at the baseline had a 6.9-fold higher OR (95% CI; 5.4–9.0) for converting to AX than HCs converting to AX, meaning the risk of anxiety conversion was high in the STA group. STA was an at-risk state for AX. Furthermore, among the 2266 respondents with baseline STA, 13.5% reported one or more episodes of AX since baseline, 12 times higher than the rate of 1.1% observed among the 9290 HCs respondents at baseline.

### 3.2. Factors Differentiating Those With STA From HCs at Baseline

We identified factors at baseline that distinguished individuals with STA from HCs. Differences in variables between baseline STA and HCs are detailed in Table S6. Figure S1 and Table S7 illustrated the significant results of the logistic analysis and revealed that sleep disturbance, level of behavioral inhibition, depressive symptoms, somatic problem, ADHD severity, degree of sluggish cognitive tempo, oppositional defiance, and family conflict were statistically significant risk factors (OR >1, *ps*  < 0.01). And sensation seeking was statistically significant protective factors (OR <1, *p* < 0.001).

### 3.3. Predictors of Developing an AX

**Figure 2:**
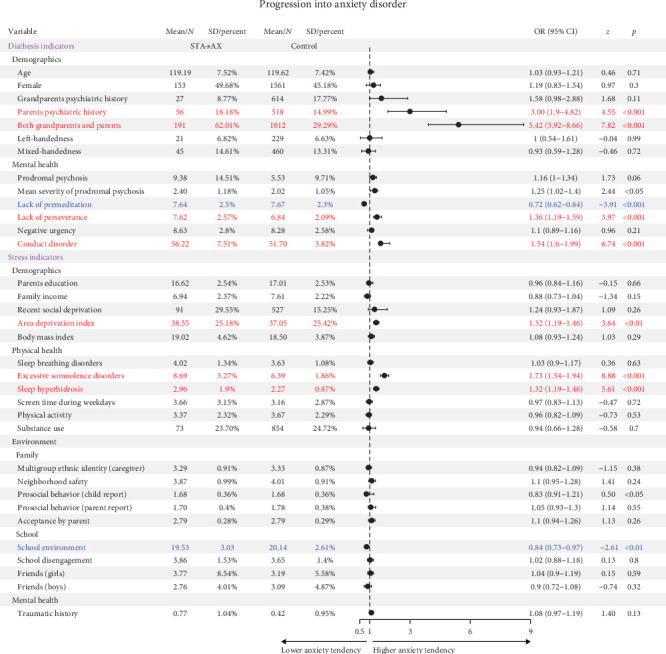
Association of progression into anxiety disorder with significant risk and protective factors illustrated through forest plot. The forest plot represents risk and protective factors of progression into AX. Red refers significant risk factors; blue refers significant protective factors. CI, confidence interval; OR, odds ratio.

Among 786 eligible STA participants, 308 participants progressed to AX. Logistic regression revealed psychiatric family history, lack of perseverance, conduct disorder symptoms, and area deprivation as statistically significant risk factors for AX development (OR >1, *ps*  < 0.001), while lack of premeditation and positive school environment emerged as statistically significant protective factors (OR <1, *ps*  < 0.01) (Figure 2, Table 1; Table S8).

### 3.4. Predictors of Persistent STA

**Figure 3:**
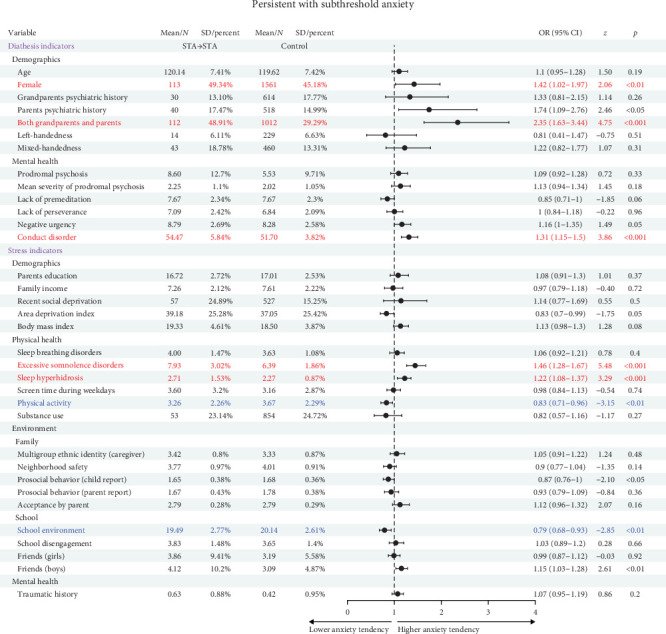
Association of remain subthreshold anxiety with significant risk and protective factors illustrated through forest plot. The forest plot represents risk and protective factors of remain STA. Red refers risk factors, which have higher anxiety tendency; blue refers protective factors, which have lower anxiety tendency. CI, confidence interval; OR, odds ratio.

Among 786 eligible STA participants, 229 participants chronically remained in STA during 3-year follow-up. Logistic regression analysis identified several statistically significant predictors (Figure 3, Table 1, and Table S9): female sex, psychiatric family history, and conduct disorder symptoms predicted persistent STA (OR >1, *ps*  < 0.01); Increased physical activity and a positive school environment were protective factors (OR <1, *ps*  < 0.01).

### 3.5. Predictors of Remission From STA

**Figure 4:**
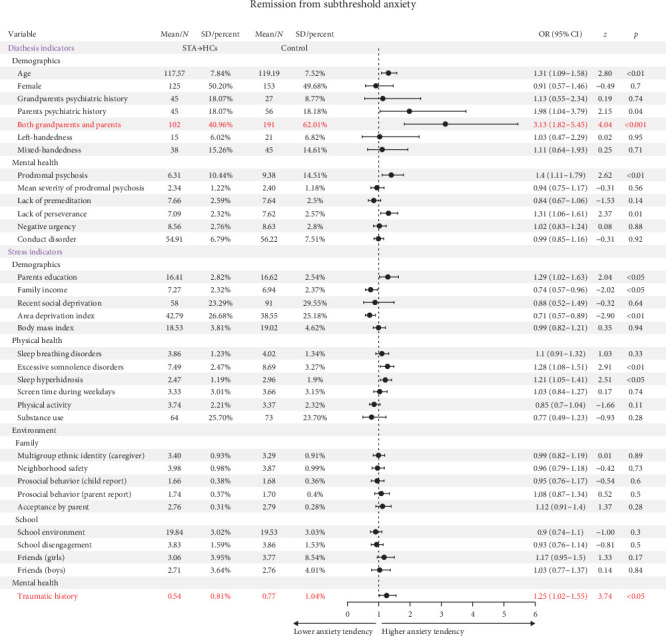
Association of remission from subthreshold anxiety with significant risk and protective factors illustrated through forest plot. The forest plot represents risk and protective factors of remission from STA. Red refers risk factors, which have higher anxiety tendency; blue refers protective factors, which have lower anxiety tendency. CI, confidence interval; OR, odds ratio.

Among 786 eligible STA participants, 249 participants remitted from STA and maintained HCs. Logistic regression analysis demonstrated statistically significant predictors of remission: Absence of psychiatric family history and absence of traumatic history (OR >1, *ps*  < 0.05) (Table 1). Complete statistical details are presented in Table S10 and Figure 4.

### 3.6. Replicated Predictors

The demonstrated predictors were replicated when using diagnostic criterion based on KSADS–5 (Tables S11–S14). The predictors were consistent in both CBCL and KSADS–5 assessment, including sleep disturbance, behavioral inhibition, somatic problem, ADHD, oppositional defiance, and sensation seeking from baseline STA versus HCs. In the three models of progression to AX, maintenance of STA, and remission from STA, a family history of psychiatric disorders was found to be a risk factor, regardless of whether it was assessed using the CBCL or KSADS. Overall, family psychiatric history showed the most consistent and robust association with high anxiety symptoms.

## 4. Discussion

This is the first study to utilize the diathesis-stress model for examining the factors influencing the development of STA in a large sample of early adolescents. Adolescents with STA showed a 6.9-fold higher risk of developing AX than baseline HCs in a 3-year period. This finding confirms that STA is an at-risk state for AX. At baseline, we identified key factors that could distinguish between STA and HCs: Depressive symptoms, somatic issues, ADHD symptoms, degree of sluggish cognitive tempo, oppositional defiance symptoms, level of behavioral inhibition, and sensation seeking as diathesis indicators; sleep disturbances and family conflict as stressors. Compared to HCs, the pivotal risk factors for progression from STA to AX were lack of perseverance and area deprivation, with females being more likely to maintain STA. Protective factors for a favorable prognosis of STA included the absence of a traumatic history, lack of premeditation, increased physical activity, and a positive school environment.

### 4.1. Shared Factors Associated With Anxiety Symptoms

Previous research consistently revealed that comorbid mental disorders (e.g., depression, somatic diseases, ADHD, sluggish cognitive tempo, oppositional defiance, and behavioral inhibition) were associated with unfavorable course of AX [11, 33–37]. From a cognitive–behavioral perspective, sluggish cognitive tempo constitutes a cognitive vulnerability characterized by negative self-schemas and impaired attentional control. These inherent diatheses interact with environmental stressors through maladaptive cognitive processes (e.g., overgeneralization of failures and catastrophizing of minor setbacks), which amplify emotional distress and manifest as anxiety symptoms [38]. Shared pathogenic mechanisms underlie the high comorbidity between oppositional defiance and AXs [39]. The current conceptualization of sensation seeking incorporates two aspects: (1) a tendency to enjoy and pursue activities that are exciting, and (2) an openness to trying new experiences that may or may not be dangerous. High scorers enjoy taking risks and engaging in dangerous activities, whereas low scorers avoid risk and danger [40]. Sensation seekers are prone to risk-taking, which may lower anxiety in the face of danger. Their readiness to confront challenges rather than avoid risks could alleviate anxiety. Insomnia, nightmares, or nocturnal panic attacks are prominent features of sleep disturbance that spans across many AX [41]. The presence of family conflict indicates the detrimental impact of unfavorable family environments on the mental health of adolescents in STA. Family conflict during early adolescence has a unique impact on individual development. Although family conflict can also lead to similar emotional and behavioral problems in adulthood, early adolescents are at a critical period of psychological and physiological development, making these impacts potentially more profound. For example, one research showed that parent relationship factors such as marital distress and separation, interpersonal violence, and conflict were associated with child psychopathology, leading to concurrent anxiety in children living in high-conflict environments [42].

Additionally, the results pertaining to the three patterns also included similar factors, for example, family psychiatric history (assessed broadly across multiple psychiatric conditions), conduct disorder symptoms, and a positive school environment. Adolescents with psychiatric family history were more likely to transition from STA to AX, remain in STA, or have difficulty remitting to HC status, representing a nonmodifiable risk factor [43]. While family studies have demonstrated significant familial co-aggregation of DSM-IV AX [44], our findings suggest that general familial psychiatric vulnerability, beyond just AXs, may influence adolescent anxiety trajectories. The presence of symptoms of conduct disorder is a vulnerability factor for the development of anxiety symptoms in children and adolescents [45]. At school, students are confronted with a larger and more complex social environment involving multiple teachers, moving between classes, and needing to function more autonomously. These challenges, combined with the increasing importance of peer relationships during this developmental stage, can lead to heightened anxiety and a desire to retreat to the security of home [46]. In contrast, adults, with more mature coping skills and established social networks, are generally less vulnerable to such environmental stressors.

Targeting modifiable factors like positive school environments, family support, and sleep quality can alleviate adolescent anxiety. Schools can provide professional psychological counseling and create peer support groups that allow students to share and support each other to help students identify and manage their anxiety. Therefore, modifiable transdiagnostic features common to insomnia could serve as important targets for AX interventions. A family environment characterized by support, understanding, and acceptance can cultivate a positive self-image, stress resilience, and anxiety prevention and remission. Ensuring regular sleep and bedtime relaxation techniques may enhance sleep quality and ease anxiety symptoms.

### 4.2. Specific Risks for Developing AX

First, our findings show that being in an at-risk state (STA) is strongly associated with an increased risk of developing an AX, as indicated by a high OR. This provides further confirmation that there is a high level of continuity among anxiety symptoms. A study inferred that there is an underlying transdiagnostic scaffolding in anxiety and related disorders, which may then have additional features specific to certain complications, comorbidities, subtypes, or even idiosyncratic differences (i.e., individual difference factors) [47]. From the transdiagnostic perspective, STA and AX may share common psychological and biological mechanisms, such as deficits in emotion regulation, cognitive biases (e.g., excessive worry), and dysregulation of the neuroendocrine system. These shared features may represent potential mechanisms underlying the observed association between STA and AX, though longitudinal studies are needed to test their causal role in progression [48]. By identifying the transdiagnostic mechanisms in STA, potential risks of AX can be detected early, allowing for early intervention to prevent the progression to full-blown AX.

Additionally, we identified factors related to transition AX, including psychiatric family history, lack of perseverance, symptoms of conduct disorder, and area deprivation. Lack of premeditation and a positive school environment were protective factors against developing AX. Lack of perseverance and area deprivation were identified as pivotal for the progression from STA to AX. Previous research showed that lack of premeditation refers to the low tendency to think and reflect on the consequences of an act before engaging in that act [40]. Lack of premeditation may constitute an endogenous protective mechanism that naturally attenuates anxiety-maintaining processes. By curtailing prolonged anticipation of adverse outcomes, this trait disrupts the formation of catastrophizing thought patterns while reducing cognitive resource allocation to threat appraisal [49]. Thus, the lack of premeditation might serve a protective function by mitigating such excessive cognitive evaluations. Lack of perseverance, defined as the inability to remain focused on a task, represents a broader trait reflecting deficits in conscientiousness. While this trait may contribute to anxiety development through cognitive energy depletion (e.g., negative automatic thoughts like “I can't handle this” disrupting sustained effort) [27], we should also consider potential reverse causality. Heightened anxiety symptoms could themselves impair task persistence by exacerbating attentional control deficits and increasing cognitive load [50]. Emotion-driven impulsivity reflects a core CBT tenet where heightened affective states disrupt cognitive control systems [51]. This manifests as: (1) impaired delay discounting that preferentially weights immediate emotion regulation over long-term goals, and (2) regulatory failures particularly evident during AX transitions, where automatic action tendencies override executive functioning. However, chronic anxiety may also amplify these effects through a feedback loop;where anxiety-induced hypervigilance depletes executive resources, thereby increasing susceptibility to impulsive responses [52]. These bidirectional relationships suggest the observed associations may reflect both predisposing factors and consequences of anxiety symptomatology. ADI is a marker of socioeconomic disadvantage, which may predict the development of worsening symptoms of anxiety [53].

Therefore, considering lack of premeditation as a modifiable diathesis factor, it is advisable for adolescents with STA to reduce rumination and excessive worry, which can help alleviate anxiety symptoms.

### 4.3. Specific Risks for Maintaining and Remission From STA

First, adolescents with STA, as an at-risk state for AX, require future preventative interventions for reducing the risk of subclinical or higher levels of anxiety. Additionally, we identified factors related to the persistence of STA, including female sex, psychiatric family history, and symptoms of conduct disorder. The group that maintained STA experienced fewer risk factors because they had a lower baseline level of risk factors that contributed to the stability of their condition rather than a worsening or transition to AX. Females with STA were prone to persistent anxiety symptoms. The specific protective factor was increased physical activity. Remission was more likely among those without a traumatic history. A cohort study found that female adolescents had higher rates of AX during puberty [54]. Early adolescence is a critical period for an individual's physical and psychological development. Current literature demonstrates physical activity's protective role in adolescent mental health [55], including psychosocial benefits of sports participation [56]. However, anxiety-related symptoms may concurrently reduce physical activity levels through behavioral inhibition pathways, suggesting bidirectional rather than purely unidirectional associations. Another study demonstrated that youth who had experienced multiple traumatic events exhibited a high level of anxiety symptoms [57].

Identifying physical activity and traumatic experiences as indicators for alleviating STA may aid in the formulation of targeted prevention strategies. Individuals experiencing STA can select appropriate forms of exercise and attempt to establish a weekly workout plan, facilitate the formation of habits, and promote physical and psychological remission. While stress, especially acute traumatic events, is a significant risk factor for AX, not everyone affected develops the disorder, highlighting the concept of “stress resilience” [58]. Resilience is defined as the ability to bounce back after an adversity or life event that was traumatic and life-changing. It is a factor that is a unique psychopathological construct as it is a biopsychosocial factor which determines an individual's response to an illness and recovery from the same. Resilience is a human capacity to adapt swiftly and successfully to stress and to revert to a positive state [59]. Previous study has showed that resilience training was acceptable to trauma-exposed individuals with varying types of subthreshold distress [60]. Therefore, for STA individuals with traumatic history, resilience-based therapy can be employed.

### 4.4. Clinical Implications

Our research highlights the need for early identification and intervention in addressing anxiety symptoms during adolescence. Preventive measures may be necessary to reduce anxiety levels and their persistence, especially during early adolescence, a critical period for developing autonomy and resilience. Screening and addressing subclinical anxiety levels are crucial for preventing mental health issues in early adolescents. For clinical practice, our results advocate for a stepped-care approach: (1) universal screening using validated tools, (2) brief resilience-building interventions for subclinical cases, and (3) specialized therapy for those meeting diagnostic thresholds. Notably, adolescents with high anxiety levels showed particular responsiveness to environmental modifications, indicating that school/family-based interventions should be prioritized for these high-risk adolescents. Identifying modifiable risk and protective factors in anxiety development can guide prevention efforts. Community interventions focusing on enhancing protective factors and reducing risks (like resilience-based therapy and increased sports activity) may play a vital role in preventing anxiety symptoms among early adolescents.

### 4.5. Limitations

In the ABCD study, participant attrition led to a reduced sample size. The use of the CBCL self-report, with its potential for bias and variability, further narrowed our subject pool as we applied strict selection criteria to ensure accurate mental health assessments over 3 years. Moreover, an important limitation of our study is our analysis was exploratory in nature that tested for baseline predictors. While our findings indicate that certain variables are associated with the prognosis of anxiety symptoms, they do not establish causality. The identified predictors are early risk indicators for anxiety in adolescents, but the underlying causal mechanisms through which these factors affect outcomes need further investigation. Additionally, the stability or instability in symptom reports may not clearly relate to actual symptoms and diagnoses. This ambiguity is due to the fact that the test–retest reliability of the reporters could influence the results. Furthermore, the KSADS used in this study was administered via computer rather than in a face-to-face format. This method may impact participants' responses and the accuracy of their reports. Nonetheless, the computer-based version of KSADS still provides reliable diagnostic information and serves a dual verification function in our analysis. A limitation of our study is the low Kappa value between CBCL and KSADS, likely due to differences in reporting sources (parents vs. physicians), which may affect the agreement between the two measures. However, we consistently identified the same risk and protective factors using both approaches. Finally, our statistical analysis did not capture the interactive or cascading effects of these factors in predicting risk, which may not accurately reflect the real-world dynamics of the diathesis-stress model. Therefore, future research should integrate advanced statistical methodologies like interaction/mediator analyses to clarify predisposition-stressor dynamics in the diathesis-stress model, boosting precision in STA risk prediction.

## 5. Conclusions

Our study is the first to examine risk and protective factors of STA transition in early adolescence, including diathesis-stress-related variables, and measure the prevalence and longitudinal outcomes of STA. STA was a common and varied condition in early life. STA is a priori risk that warrants a focus on prevention. Our study outlines modifiable factors like healing traumatic experiences, increased physical activity, and the enhancement of school and family environments that may help prevent STA from worsening. However, caution is needed in interpreting these findings. Further research is essential to confirm whether these factors can be effectively targeted to prevent anxiety symptoms in at-risk adolescents.

## Figures and Tables

**Table 1:** Significant risk and protective factors of three patterns.

Patterns	Variable	Odds ratio	95% CIs		*β*	*z*	Wald statistic	*p*
			Lower	Upper				
STA → AX vs. HCs	Diathesis indicators							
	Demographics							
	Parents' psychiatric history	3.00	1.90	4.82	1.10	4.55	19.81*⁣*^*∗∗∗*^	<0.001
	Grandparents and parents' psychiatric history	5.42	3.92	8.66	1.69	7.82	64.60*⁣*^*∗∗∗*^	<0.001
	Mental health							
	Lack of premeditation	0.72	0.62	0.84	−0.32	−3.91	14.20*⁣*^*∗∗*^	<0.001
	Lack of perseverance	1.36	1.19	1.59	0.31	3.97	16.20*⁣*^*∗∗*^	<0.001
	Conduct disorder	1.54	1.60	1.99	0.43	6.74	51.11*⁣*^*∗∗∗*^	<0.001
	Stress indicators							
	Demographics							
	Area deprivation index	1.32	1.19	1.46	0.28	3.64	13.27*⁣*^*∗∗*^	<0.001
	Physical health							
	Excessive somnolence disorders	1.73	1.54	1.94	0.55	9.11	83.87*⁣*^*∗∗∗*^	<0.001
	Sleep hyperhidrosis	1.32	1.19	1.46	0.28	5.35	29.4*⁣*^*∗∗∗*^	<0.001
	Environment							
	School environment	0.84	0.73	0.97	−0.18	−2.61	6.18*⁣*^*∗*^	<0.01

STA → STA vs. HCs	Diathesis indicators							
	Demographics							
	Female	1.42	1.02	1.97	0.35	2.06	4.43*⁣*^*∗*^	<0.01
	Grandparents and parents' psychiatric history	2.35	1.63	3.44	0.85	4.75	20.11*⁣*^*∗∗∗*^	<0.001
	Mental health							
	Conduct disorder	1.31	1.15	1.5	0.27	3.86	16.31*⁣*^*∗∗*^	<0.001
	Stress indicators							
	Physical health							
	Excessive somnolence disorders	1.46	1.28	1.67	0.38	5.48	32.92*⁣*^*∗∗∗*^	<0.001
	Sleep hyperhidrosis	1.22	1.08	1.37	0.2	3.29	10.96*⁣*^*∗*^	<0.001
	Physical activity	0.83	0.71	0.96	−0.19	−3.15	8.23*⁣*^*∗*^	<0.01
	Environment							
	School environment	0.79	0.68	0.93	−0.23	−2.85	8.76*⁣*^*∗*^	<0.01

STA → HCs vs. STA → AX	Diathesis indicators							
	Demographics							
	Grandparents and parents' psychiatric history	3.13	1.82	5.45	1.14	4.04	16.69*⁣*^*∗∗*^	<0.001
	Stress indicators							
	Mental health							
	Traumatic history	1.25	1.02	1.55	0.22	3.74	8.37*⁣*^*∗*^	<0.05

*⁣* ^*∗*^ *p* < 0.05 following Bonferroni correction.

*⁣* ^*∗∗*^ *p* < 0.01 following Bonferroni correction.

*⁣* ^*∗∗∗*^ *p* < 0.001 following Bonferroni correction.

## Data Availability

The data used in the preparation of this article were obtained from the Adolescent Brain Cognitive Development (ABCD) Study (https://abcdstudy.org/), held in the NIMH Data Archive (NDA).

## Supporting Information

Supporting Information Supporting information includes supporting method, Supporting results, Tables S1–S14 and Figures S1 of the nonprimary results of this study.
