# Supplementary material for: Identifying Risk and Protective Factors Impacting the Clinical Outcomes of Subthreshold Anxiety in Early Adolescents: Insights From the ABCD Study
Source: Depress Anxiety. 2025 Jun 25;2025:6514030. doi: 10.1155/da/6514030 (PMC12221549; doi:10.1155/da/6514030)
Supplement: Supporting Information — includes supporting method, Supporting results, Tables S1–S14 and Figures S1 of the nonprimary results of this study. [file 6514030.f1.docx]

# Supplementary Information

## Identifying Risk and Protective Factors Impacting the Clinical Outcomes of Subthreshold Anxiety in Early Adolescents: Insights from the ABCD Study

Chen Keyin^1^, Li Qian^1^, Zhang Jiayuan^1^, Niu Lijing^1^, Dai Haowei^1^, Peng lanxin^1^, Wang Xingqin^2^, Ma Qing^3^**^#^**, Zhang Ruibin ^1,4,5^**^#^**

^1^ Laboratory of Cognitive Control and Brain Healthy, Department of Psychology, School of Public Health, Southern Medical University, Guangzhou, PRC China

^2^ Department of Neurosurgery, Institute of Brain Diseases, Nanfang Hospital of Southern Medical University, Guangzhou, PRC China

^3.^ School of Psychology and Cognitive Science, East China Normal University, Shanghai, China

^4.^ Guangdong-Hong Kong-Macao Greater Bay Area Center for Brain Science and Brain-Inspired Intelligence, Guangdong-Hong Kong Joint Laboratory for Psychiatric Disorders, Guangdong Basic Research Center of Excellence for Integrated Traditional and Western Medicine for Qingzhi Diseases, Guangzhou, PRC China

^5.^ Department of Psychiatry, Zhujiang Hospital, Southern Medical University, Guangzhou, PRC China

^#^ Correspondence and request for materials should be addressed to:

Ma Qing, School of Psychology and Cognitive Science, East China Normal University, Shanghai, China, China (e-mail: [qingmascnu@gmail.com](mailto:qingmascnu@gmail.com))

Ruibin Zhang, Department of Psychology, School of Public Health, Southern Medical University (e-mail: [ruibinzhang@foxmail.com](mailto:ruibinzhang@foxmail.com))

Contents

[Supplementary Information 1](#_Toc199438564)

[Identifying Risk and Protective Factors Impacting the Clinical Outcomes of Subthreshold Anxiety in Early Adolescents: Insights from the ABCD Study 1](#_Toc199438565)

[Supplementary Methods 5](#_Toc199438566)

[Predictors 5](#_Toc199438567)

[Concordance analysis 8](#_Toc199438568)

[Measures 8](#_Toc199438569)

[Anxiety Disorder 8](#_Toc199438570)

[Subthreshold Anxiety 8](#_Toc199438571)

[Recovery from Subthreshold Anxiety 8](#_Toc199438572)

[Supplementary Results 9](#_Toc199438573)

[Prevalence, natural course and longitudinal outcome 9](#_Toc199438574)

[Supplementary Tables 10](#_Toc199438575)

[Table S**1** List of measures, measure characteristics, and psychometric information. 10](#_Toc199438576)

[Table S**2** Measures of demographics, physical health, mental health and environment. 16](#_Toc199438577)

[Table S**3** Differences in variables for four groups (N=4241). 21](#_Toc199438578)

[Table S**4** Differences in baseline variables for eligible participants and excluded subthreshold anxiety groups (N=2421). 24](#_Toc199438579)

[Table S**5** Multinomial logistic regression analysis (STA→HCs, STA→STA, STA→AX, and HCs). 26](#_Toc199438580)

[Table S**6** Differences in variables for baseline subthreshold anxiety and healthy controls (N=11556). 30](#_Toc199438581)

[Table S**7** Baseline factors differentiating subthreshold anxiety from healthy controls (CBCL). 32](#_Toc199438582)

[Table S**8** Risk and protective factors of developing an anxiety disorder (CBCL). 34](#_Toc199438583)

[Table S**9** Risk and protective factors of remained subthreshold anxiety (CBCL). 36](#_Toc199438584)

[Table S**10** Risk and protective factors of remission from subthreshold anxiety (CBCL). 38](#_Toc199438585)

[Table S**11** Baseline factors differentiating subthreshold anxiety from healthy controls (KSADS-5). 40](#_Toc199438586)

[Table S**12** Significant risk and protective factors of progression into anxiety disorder (KSADS-5). 42](#_Toc199438587)

[Table S**13** Significant risk and protective factors of remained subthreshold anxiety (KSADS-5). 43](#_Toc199438588)

[Table S**14** Significant risk and protective factors of remission from subthreshold anxiety (KSADS-5). 44](#_Toc199438589)

[Figure S**1** Association of higher anxiety symptoms with significant risk and protective factors illustrated through forest plot (Baseline subthreshold anxiety vs healthy controls). 45](#_Toc199438590)

[Data availability for production 46](#_Toc199438591)

[COI statement for production 46](#_Toc199438592)

[Funding statement for production 46](#_Toc199438593)

[Supplementary References 47](#_Toc199438594)

# Supplementary Methods

## Predictors

31 putative predictors were selected from demographics, physical health, mental health and environment variables and subdivided into diathesis and stress related variables (Chen et al., 2025).

Among the diathesis related variables; there are mainly demographics and mental health variables. Among the demographics related variables: it is an extensive demographic questionnaire composed primarily of items and questions from the PhenX toolkit (Stover, Harlan, Hammond, Hendershot, & Hamilton, 2010), which we used age (age in months at the time of the interview), gender (sex at birth) and handedness. Family history of psychological problems include: alcohol problem, drug use problem, depression problem, mania problem, parents visions of others spying/plotting problem, parents trouble holds job/fights/police problem, parents nerves/nervous breakdown problem, parents been to a doctor or counselor due to emotional/mental problem, parents hospitalized due to emotional/mental problem and parents attempted or committed suicide. Using psychiatric histories from the 2 generations—grandparents (generation 1 [G1]) and parents (generation 2 [G2])—we formed a 4-category anxiety risk variable, reflecting how many prior generations were affected with anxiety: (1) neither G1 nor G2 (G1−/G2−; lowest risk assumed), (2) only G1 (G1+/G2−), (3) only G2 (G1−/G2+), and (4) both G1 and G2 (G1+/G2+; highest risk assumed). Mental health variables: The behavioral inhibition system (BIS) subscale includes 7 questions to assess levels of behavioral inhibition. UPPS-P Impulsive Behavior Scale (UPPS-P) is a 59-item questionnaire assessing impulsive personality traits. This scale includes the following five subscales: negative urgency (NU), lack of premeditation (LPM), lack of perseverance (LPS), sensation seeking (SS), and positive urgency (PU) (Whiteside & Lynam, 2001). Each subscale has 10–14 items and a higher score represents a greater level of impulsivity. ABCD asks the parents to report annually starting at baseline about the youth’s behavior using the CBCL, which includes depression, somatic problems, ADHD, oppositional defiance, conduct disorder and sluggish cognitive tempo (t-scores). At baseline and likely for the next several longer bi-annual follow-ups that coincide with imaging, ABCD have the parent complete almost all of the modules in the KSADS-5. Prodromal Psychosis is assessed by Prodromal Psychosis Scale (PPS), and we used the severity score sum and mean endorsed severity score.

Among the stress related variables; there are demographics, physical health, environment and mental health variables. Demographics: Parent education is based on the number of years of education (range 0-21 years). Family income is based on questions from the General Social Survey (Smith T.W., 1972–2012), in order to assess income-to-needs and overall income. Area Deprivation Index (ADI) is a measure of socioeconomic disadvantage at the neighborhood level. It is based on 17 metrics derived from Census data describing poverty, education, employment, and housing quality, with a higher score indicating greater neighborhood deprivation (K, 2003). Social deprivation index (SDI) is a composite measure of seven demographic characteristics collected in the American Community Survey (ACS): percent living in poverty, percent with less than 12 years of education, percent single-parent households, the percentage living in rented housing units, the percentage living in the overcrowded housing unit, percent of households without a car, and percentage nonemployed adults under 65 years of age (Butler, Petterson, Phillips, & Bazemore, 2013). Physical health: The height and weight of each subject was measured up to three times and averaged. BMI was calculated by multiplying 703 by mean weight in pounds divided by mean height in inches squared (Dennis, Manza, & Volkow, 2022). Screen time is the time of a youth’s use of various electronic devices, there developed a brief assessment obtaining parent’s report of their youth’s use of visual media based on the work of Sharif, Wills and Sargent (I. Sharif, T. A. Wills, & J. D. Sargent, 2010). This measure includes two questions on the overall amount of time that the youth spend using visual media, one about a typical weekday and one about a typical weekend day. A study found associations between increased screen time, particularly computer use, and a small increased risk of anxiety and depression (Khouja et al., 2019). Physical activity was measured by self-report as the number of days in the week prior to the interview that each subject was physically active for a total of at least 60 minutes per day. This data was collected via questionnaire from the Youth Risk Behavior Survey as part of the ABCD Study’s baseline physical health battery (Barch et al., 2018). We selected the Sleep Disturbance Scale for Children (SDSC) for its validity for this age period, comprehensive screening for a variety of sleep disturbance types, and brevity. Developed and validated for school aged children and adolescents, the SDSC (O. Bruni et al., 1996) is a 26-item Likert-type rating scale administered to a parent. The six scales are: (1) disorders of initiating and maintaining sleep; (2) sleep breathing disorders; (3) disorders of arousal or nightmares; (4) sleep wake transition disorders; (5) disorders of excessive somnolence; and (6) sleep hyperhidrosis, which are typically associated with neurological dysfunction or pathological changes. Adolescent substance use is measured using self-reported substance use over the past six months involving tobacco, alcohol, caffeine, marijuana, and so on. Environmental factors include cultural, family and social environment. Ethnic identity is assessed in the ABCD study with the Multigroup Ethnic Identity Measure-Revised (Phinney & Ong, 2007). Parents self-categorize their ethnic group identity via an open-ended item on the MEIM-R. The measure ABCD used was taken from the PhenX Toolkit, and was derived from the “Safety from Crime” items of scales assessing neighborhood characteristics (Echeverria, Diez-Roux, & Link, 2004). The PhenX measure consists of three statements assessing feelings about safety and presence of crime in the respondent’s neighborhood, including feeling safe walking in one’s neighborhood, violence in the neighborhood, and crime in the neighborhood. School performance may serve as early indicator of challenges or as a predictor of future resilience or impairment. In the ABCD protocol, the 12-item Inventory for School Risk and Protective Factors (SRPF) was selected to assess three dimensions of this general concept of a child’s connectedness to his/her school: school teacher and classroom environment, personal involvement in school, and alienation from academic goals. The SRPF inventory is scored for these three scales. The Acceptance Scale, a subscale of the Child Report of Behavior Inventory (CRPBI) (Schaefer, 1965), is used to assess youth’s perceptions of caregiver warmth, acceptance, and responsiveness. The scale was shortened from 10 to five items and youth respond to items describing caregivers’ behaviors on a three-point scale indicating the extent to which their caregiver’s behavior fits the descriptor on a particular item indicating warmth or acceptance. The ABCD protocol is utilizing the 9 −item Family Conflict subscale of the Moos Family Environment Scale (FES) for the baseline protocol, with repeated assessment planned every 2 years. The measure is widely used, has a binary response self-report format, and is also a part of the PhenX Toolkit. For scoring, each true/false item is assigned a value of 0 or 1, with appropriate reverse coding for those 4 items that negate conflict instead of describing the direct presence of it (e.g., family members hardly ever lose their temper). Higher scores indicate a more-conflictual family environment. Prosocial behavior (e.g., being nice, helping, caring) is assessed using the Prosocial Behavior Scale, a subscale from the “Strengths and Difficulties Questionnaire” (R. Goodman, Meltzer, H., & Bailey, V, 1998). The original subscale has 5 items, and ABCD retained three items with the highest factor loadings. Both parents and youth report on the youth’s prosocial behavior (e.g., being considerate of other people’s feelings, often offering to help others). They rate these behaviors over the past 6 months on a three-point scale (“0 = Not True” to “2 = Certainly True”). A study found that higher levels of social anxiety are negatively associated with prosocial behaviors (Yang et al., 2024). ABCD assess information about friendships from the youth’s perspectives, as friendship quantity and quality may be both an early indicator of interpersonal function and a predictor of later outcome. Mental health: traumatic experiences are assessed as part of the parent-report post-traumatic stress disorder module of the KSADS-5 (num of traumatic experiences).

# Concordance analysis

## Measures

The prognosis of subthreshold anxiety (STA) was based on the three outcome variables according to Parent Diagnostic Interview for DSM-5 (The Diagnostic and Statistical Manual) Full (The Kiddie Schedule for Affective Disorders and Schizophrenia, KSADS-5): ‘anxiety disorder (AX)’, ‘STA’, and ‘recovery from STA’.

### Anxiety Disorder

KSADS-5 was also applied to identify adolescents with anxiety symptoms. According to KSADS-5 (Zimmerman, Thompson, Diehl, Balling, & Kiefer, 2020), AX is defined as having 5 items at a severe level (e.g., feeling keyed up or tense, feeling restless, difficulty concentrating because of worry, fear that something awful might happen, and feeling that one might lose control), duration of 6 months and some level of functional disability (e.g., impaired concentration due to worry interferes with some activities).

### Subthreshold Anxiety

According to KSADS-5, the subthreshold anxiety group was defined as individuals who met at least two criteria, including the core symptoms of each anxiety disorder, but did not fulfill the diagnostic criteria (Bosman et al., 2019).

### Recovery from Subthreshold Anxiety

Healthy control (HC) is defined as having 0/1 symptom in KSADS-5 (Zimmerman et al., 2020).

#

# Supplementary Results

## Prevalence, natural course and longitudinal outcome

Longitudinal studies have clear advantages, particularly when they are based on representative samples assessed throughout the core high-risk period of first onset and subsequent potential periods of chronic illness. Based on the three-year follow-up assessed at baseline, among 11868 participants, 2.6% experienced an AX, 19.1% experienced a STA and 78.3% experienced no anxiety symptoms. At one-year follow-up, among 11207 participants, 2.9% experienced an AX, 19.0% experienced a STA and 78.1% experienced no anxiety symptoms. At two-year follow-up, among 8085 participants, 2.3% experienced an AX, 20.1% experienced a STA and 77.6% experienced no anxiety symptoms. At three-year follow-up, among 6133 participants, 2.3% experienced an AX, 23.2% experienced a STA and 74.4% experienced no anxiety symptoms (Figure 1C).

# Supplementary Tables

## Table S**1** List of measures, measure characteristics, and psychometric information.

|  | **Variable Name** | **Variable Description** | **Measure** | **Psychometric information** | **Empirical evidence** |
| --- | --- | --- | --- | --- | --- |
| *Diathesis-related variables* |  |  |  |  |  |
| Demographic variables | age | age in months at the time of the interview | PhenX toolkit (Stover et al., 2010) |  |  |
|  | sex | sex at birth | PhenX toolkit |  |  |
|  | psychiatric family histories | Family history of psychological problems:  alcohol problem, drug use problem, depression problem, mania problem, parents visions of others spying/plotting problem, parents trouble holds job/fights/police problem, nerves/nervous breakdown problem, parents been to a doctor or counselor due to emotional/mental problem, parents hospitalized due to emotional/mental problem, parents attempted or committed suicide | Modification of the Family History Assessment from the National Consortium on Alcohol and NeuroDevelopment in Adolescence (NCANDA) (Brown et al., 2015) |  |  |
|  | handedness |  | PhenX toolkit |  |  |
| Mental health variables | behavioral inhibition |  | Child Behavioral Inhibition & Behavioral Activation Scales from PhenX (Jorm et al., 1998) | Cronbach's alpha was 0.76 for BIS (Jorm et al., 1998). | (Sandstrom, Uher, & Pavlova, 2020) |
|  | UPPS-P impulsive behavior scale | this scale includes five subscales: negative urgency, lack of premeditation, lack of perseverance, sensation seeking, and positive urgency | Modified UPPS-P for Children from PhenX (Whiteside & Lynam, 2001) | The internal consistency was 0.74 – 0.88 across subscales (Whiteside & Lynam, 2001). | (Jakuszkowiak-Wojten, Landowski, Wiglusz, & Cubała, 2015) |
|  | prodromal psychosis |  | Pediatric Psychosis Questionnaire − Brief Version (Loewy, Bearden, Johnson, Raine, & Cannon, 2005) | High sensitivity (90%), low specificity (49%) (Loewy et al., 2005). | (Morales-Muñoz, Palmer, Marwaha, Mallikarjun, & Upthegrove, 2022) |
|  | depression |  | Achenbach Child Behavior Check List (CBCL) (Achenbach, 2011) | The reliability was 0.94 and the validity was 0.92 (Achenbach, 2011). | (Asselmann, Wittchen, Lieb, Höfler, & Beesdo-Baum, 2014) |
|  | somatic problems |  |  |  | (Mallorquí-Bagué, Bulbena, Pailhez, Garfinkel, & Critchley, 2016) |
|  | attention-deficit/hyperactivity disorder (ADHD) |  |  |  | (Koyuncu, Ayan, Ince Guliyev, Erbilgin, & Deveci, 2022) |
|  | oppositional defiance |  |  |  | (Rowe, Costello, Angold, Copeland, & Maughan, 2010) |
|  | conduct disorder |  |  |  | (Rowe et al., 2010) |
|  | sluggish cognitive tempo |  |  |  | (Skirbekk, Hansen, Oerbeck, & Kristensen, 2011) |
| *Stress-related variables* |  |  |  |  |  |
| Demographics variables | parent education | parent highest education | PhenX toolkit |  |  |
|  | family income | total combined family income for past 12 months | PhenX toolkit |  |  |
|  | area deprivation index (ADI) |  | The Health Resources & Services Administration (HRSA) (Singh, 2003) | The reliability coefficient (α) for the index was 0.95 (Singh, 2003). |  |
|  | recent social deprivation |  | American Community Survey (ACS) (Butler et al., 2013) | The factor loadings were greater than 0.60 (Butler et al., 2013). |  |
|  | body mass index（BMI） |  | Height, Weight, BMI, and Waist Circumference from National Health and Nutrition Examination Survey (Curtin et al., 2013) |  |  |
| Physical health variables |  |  |  |  |  |
|  | screen time | screen time weekday sum | Screen Time Questionnaire for Parent (Iman Sharif, Thomas A. Wills, & James D. Sargent, 2010) | Cronbach's alpha = 0.75 (Iman Sharif et al., 2010) | (Khan, Lee, & Horwood, 2022) |
|  | physical activity | over the past 7 days | Sports and Activities Involvement Questionnaire (Brener, Collins, Kann, Warren, & Williams, 1995) | Kappas ranged from 14.5% to 91.1% (Brener et al., 1995). | (Yan et al., 2024) |
|  | sleep disturbances | this scale includes six subscales: disorders of initiating and maintaining sleep, sleep breathing disorders, disorders of arousal or nightmares, sleep wake transition disorders, disorders of excessive somnolence, sleep hyperhydrosis. | Sleep Disturbances Scale for Children (Oliviero Bruni et al., 1996) | The test/retest reliability was 0.71; the internal consistency was 0.79 (Oliviero Bruni et al., 1996). | (Chellappa & Aeschbach, 2022) |
|  | substance use | over the past 6 months | Timeline Follow-back (TLFB) (Robinson, Sobell, & Leo, 2012) | The test–retest reliability was 0.82 (Robinson et al., 2012). | (Asselmann et al., 2014) |
| Environmental variables | parents' multi-group ethnic identity |  | Multigroup ethnic identity  measure- Revised (MEIM-R) (Herrington, Smith, Feinauer, & Griner, 2016) | Reliability coefficients = 0.84 (Herrington et al., 2016). | (Bell et al., 2025) |
|  | neighborhood safety |  | Neighborhood Safety/Crime Survey Modified from PhenX | Cronbach's alpha coefficient was 0.87 (Mujahid, Diez Roux, Morenoff, & Raghunathan, 2007). | (Vane, Habhab, Corona, & Mednick, 2021) |
|  | family conflict |  | Family Conflict subscale of the Moos Family Environment Scale (FES) (Moos & Moos, 1976) | Cronbach's alpha coefficient ranged from 0.68 to 0.86, with test-retest reliabilities of 0.60–0.85 (Lanz & Maino, 2014). | (Rapee, 2012) |
|  | prosocial behavior |  | Prosocial Behavior subscale from Strengths and Difficulties  Questionnaire (R. Goodman, Meltzer, & Bailey, 1998) | Cronbach's alpha coefficient was 0.82 (R. Goodman et al., 1998). | (Yang et al., 2024) |
|  | acceptance by parents |  | Acceptance Subscale from Children's Report of Parental Behavioral Inventory (CRPBI) (Schaefer, 1965) | Cronbach's alpha coefficient was 0.71 (Zucker et al., 2018). | (Rienks et al., 2025) |
|  | school environment |  | School Risk & Protective Factors Survey derived from The Communities That Care (CTC) Youth Survey (Arthur et al., 2007) | Cronbach's alpha coefficient was 0.60 (Arthur et al., 2007). | (Blöte, Miers, Heyne, & Westenberg, 2015) |
|  | school disengagement |  |  | Cronbach's alpha coefficient was 0.21 (Arthur et al., 2007). | (Blöte et al., 2015) |
|  | close friends | the number of close friends (girls and boys) |  |  | (Greco & Morris, 2005) |
| Mental health variables | traumatic experiences | the number of traumatic experiences | Kiddie Schedule for Affective Disorders and Schizophrenia (KSADS-5) (Kaufman et al., 1997) | Test-retest reliability kappa coefficients were 0.77 to 1.00, (Kaufman et al., 1997). The inter-rater reliability kappa value of the scale was above 0.8 (Nishiyama et al., 2020). | (Seok et al., 2020) |

## Table S**2** Measures of demographics, physical health, mental health and environment.

Field names are the column names used in the original ABCD curated data. For those measures that used multiple data fields, notes are provided for the methods of creating them.

| **File Name** | **Variable Name** | **Variable Description** | **Items** | **Value** | **Data type** | **Num of missing** | **Collect from** |
| --- | --- | --- | --- | --- | --- | --- | --- |
| **Demographics** | | | | | | | |
|  | interview_age | Age | 1 | (months) | Integer | NA |  |
|  | gender | Gender | 1 | F = female; M = male | String | NA |  |
| abcd_ehis01 | ehi_y_ss_scoreb | Handedness score | 1 | 1 = right-handed; 2 = left-handed; 3 = mixed-handed | Integer | NA |  |
| abcd_rhds01 | reshist_addr1_adi_perc | Area deprivation index (ADI): national percentiles | 1 |  | Float | 879 | Parents |
| pdem02 | demo_fam_exp1/2/3/4/5/6/7_v2 | Social Deprivation-7 questions | 7 | 0 = No; 1 = Yes | Integer | 135 | Parents |
| pdem02 | demo_comb_income_v2 | Total combined family income for past 12 months | 1 | 1= ≤$5,000; 2=$5,000~$11,999; 3=$12,000~$15,999; 4=$16,000~$24,999; 5=$25,000~$34,999; 6=$35,000~$49,999; 7=$50,000~$74,999;  8= $75,000~$99,999; 9=$100,000~$199,999;  10=≥$200,000. | Integer | 1018 | Parents |
| pdem02 | demo_prnt_ed_v2 | Parent highest education | 1 | 0 = Never attended/Kindergarten; 1~12=1st~12th grade; 13 = High school graduate; 14 = GED; 15 = Some college; 16 = Associate degree; 17 = Associate degree; 18 = Bachelor's degree; 19 = Master's degree; 20 = Professional School degree; 21 = Doctoral degree; | Integer | 17 | Parents |
| abcd_fhxssp01 | famhx_ss_parent_alc_p | Family history of psychological problems (first- and second-degree, biological relatives)  Overall parents alcohol problem | 10 (1=2/3/-1/-2) | 1 = none; 2 = only grandparents; 3 = only parents; 4 = both grandparents and parents have psychiatric history | Integer | 178 | Parents |
|  | famhx_ss_parent_dg_p | Overall parents drug use problem |  |  |  |  |  |
|  | famhx_ss_parent_dprs_p | Overall parents depression problem |  |  |  |  |  |
|  | famhx_ss_parent_ma_p | Overall parents mania problem |  |  |  |  |  |
|  | famhx_ss_parent_vs_p | Overall parents visions of others spying/plotting problem |  |  |  |  |  |
|  | famhx_ss_parent_trb_p | Overall parents trouble holds job/fights/police problem |  |  |  |  |  |
|  | famhx_ss_parent_nrv_p | Overall parents nerves/nervous breakdown problem |  |  |  |  |  |
|  | famhx_ss_parent_prf_p | Overall parents been to a doctor or counselor due to emotional/mental problem |  |  |  |  |  |
|  | famhx_ss_parent_hspd_p | Overall parents hospitalized due to emotional/mental problem |  |  |  |  |  |
|  | famhx_ss_parent_scd_p | Overall parents attempted or committed suicide |  |  |  |  |  |
|  | BMI | Body mass index | 1 |  | Integer | 478 |  |
| **Physical health** | | | | | | | |
| abcd_ssmty01 | stq_y_ss_weekday | Screen time weekday Sum | 1 |  | Float |  | self |
|  | stq_y_ss_weekend | Screen time weekend Sum | 1 |  |  |  |  |
| abcd_ssphp01 | sds_p_ss_dims | Disorders of Initiating and Maintaining Sleep (DIMS) SUM | 7 |  | Integer | 5 | Parents |
|  | sds_p_ss_sbd | Sleep Breathing disorders (SBD) SUM | 3 |  |  | 5 |  |
|  | sds_p_ss_da | Disorder of Arousal (DA) SUM | 3 |  |  | 5 |  |
|  | sds_p_ss_swtd | Sleep-Wake transition Disorders (SWTD) SUM | 6 |  |  | 32 |  |
|  | sds_p_ss_does | Disorders of Excessive Somnolence (DOES) SUM | 5 |  |  | 6 |  |
|  | sds_p_ss_shy | Sleep Hyperhydrosis (SHY) SUM | 2 |  |  | 5 |  |
| abcd_yrb01 | physical_activity1_y | During the past 7 days, on how many days were you physically active for a total of at least 60 minutes per day? | 1 | 0 = 0 days; 1 = 1 day; 2 = 2 days; 3 = 3 days; 4 = 4 days; 5 = 5 days; 6 = 6 days; 7 = 7 days | Integer | 28 | self |
| abcd_ysu02 | tlfb_/_use | Past 6-month detailed patterns of substance use | 53 | 0 = No; 1 = Yes | Integer |  | self |
| **Mental health** | | | | | | | |
| abcd_ptsd01 | ksads_ptsd_raw_754_p~ksads_ptsd_raw_770_p | Traumatic History (DSM-5 Interview, KSADS): trauma index (num of traumatic experiences) | 17 | 0 = No; 1 = Yes | Integer | 286 | Parents |
| abcd_mhy02 | pps_y_ss_severity_score | Prodromal Psychosis Scale: Severity Score Sum | 21 | 0 = No; 1 = Yes. Distress score | Integer | 23 | self |
|  | pps_ss_mean_severity | Prodromal Psychosis Scale: Mean Endorsed Severity Score PPS severity score | 21 | 0 = Yes but not bother (see question: pps_bother_yn); 1 = Not very bothered; 5 = Extremely bothered | Integer |  |  |
| abcd_mhy02 | upps_y_ss_lack_of_planning | UPPS-P for Children Short Form: Lack of Premeditation | 5 | 1/4 = Not at all like me; 2/3 = Not like me; 3/2 = Somewhat like me; 4/1 = Very much like me | Integer | 33 | self |
|  | upps_y_ss_lack_of_perseverance | Lack of Perseverance | 5 |  |  | 23 |  |
|  | upps_y_ss_negative_urgency | Negative Urgency | 5 |  |  | 24 |  |
|  | upps_y_ss_sensation_seeking | Sensation Seeking | 5 |  |  | 23 |  |
|  | upps_y_ss_positive_urgency | Positive Urgency | 5 |  |  | 23 |  |
| abcd_mhy02 | bis_y_ss_bis_sum | Behavioral Inhibition (BIS): Inhibition | 7 (reverse scoring: bisbas5_y) | 0=Not true; 1=Somewhat true; 2=True; 3=Very true | Integer | 37 | self |
| abcd_cbcls01 | cbcl_scr_dsm5_depress_t | Depression: CBCL DSM5 Scale (t-score) | 1 |  | Integer | 8 | Parents |
|  | cbcl_scr_dsm5_somaticpr_t | Somatic problems | 1 |  |  |  |  |
|  | cbcl_scr_dsm5_adhd_t | ADHD | 1 |  |  |  |  |
|  | cbcl_scr_dsm5_opposit_t | Oppositional defiance | 1 |  |  |  |  |
|  | cbcl_scr_dsm5_conduct_t | Conduct disorder | 1 |  |  |  |  |
|  | cbcl_scr_07_sct_t | Sluggish Cognitive Tempo (SCT) | 1 |  |  |  |  |
| **Environment** | | | | | | | |
| abcd_sscep01 | meim_p_ss_total | Caregiver: Multi-group ethnic identity (total scale) | 1 |  | Float | 690 | Parents |
| abcd_sscep01 | nsc_p_ss_mean_3_items | Caregiver: Neighbourhood safety | 1 |  | Float | 8 | Parents |
| abcd_sscey01 | srpf_y_ss_ses | Child: School risk and protective factors, Environment Subscale | 1 |  | Integer | 25 | self |
|  | srpf_y_ss_iiss | Child: School risk and protective factors, Involvement Subscale | 1 |  |  |  |  |
|  | srpf_y_ss_dfs | Child: School risk and protective factors, Disengagement Subscale | 1 |  |  |  |  |
| abcd_sscey01 | fes_y_ss_fc_pr | Conflict Subscale from the Family Environment Scale, Sum of Youth Report: Prorated Score | 1 |  | Float | 24 | self |
| abcd_sscey01 | psb_y_ss_mean | Prosocial Behavior Subscale Mean of Youth Self Report | 1 |  | Float | 33 | self |
| abcd_sscep01 | psb_p_ss_mean | Prosocial Behavior Subscale Mean of Parent Report on Youth | 1 |  | Float | 24 | Parents |
| abcd_sscey01 | crpbi_y_ss_parent | Acceptance Subscale Mean of Report by Parent Completing Protocol by youth | 1 |  | Float | 34 | self |
| abcd_ysr01 | resiliency5b_y | How many CLOSE friends that are boys do you have? | 1 | Close friends are those you like spending time with, have fun with, and trust | String | 26 | self |
|  | resiliency6b_y | How many CLOSE friends that are girls do you have? | 1 |  |  | 30 |  |

## Table S**3** Differences in variables for four groups (N=4241).

|  | | Baseline | | Follow-up | | | | | | | | |  | | |  |
| --- | --- | --- | --- | --- | --- | --- | --- | --- | --- | --- | --- | --- | --- | --- | --- | --- |
|  | | STA  (N=786) | | AX  (N=308) | | | chronic STA  (N=229) | | | C  (N=249) | | | HC  (N=3455) | | | F |
|  | |  | | STA progression into AX | | | Remained STA | | | Remission from STA | | | Remained HC | | |  |
|  |  | | | | | | | | | | | | | | | |
| **Diathesis indicators** |  | | | | | | | | | | | | | | | |
| **Demographics** | |  | |  | | |  | | |  | | |  | | |  |
|  | | Mean | S.D. | Mean | S.D. | | Mean | S.D. | | Mean | S.D. | | Mean | S.D. | |  |
| Age | | 118.95 | 7.65 | 119.19 | 7.52 | | 120.14 | 7.41 | | 117.57 | 7.84 | | 119.62 | 7.42 | | 6.61^***^ |
| Picture Vocabulary Test | | 108.18 | 17.56 | 109.00 | 19.23 | | 108.40 | 17.17 | | 106.96 | 15.67 | | 108.12 | 16.47 | | 0.65 |
| Flanker Inhibitory Control and Attention Test | | 95.28 | 13.16 | 94.79 | 13.31 | | 96.26 | 14.41 | | 94.98 | 11.68 | | 96.91 | 13.83 | | 3.28^*^ |
| Pattern Comparison Processing Speed Test | | 92.52 | 22.80 | 92.42 | 22.19 | | 93.81 | 23.98 | | 91.45 | 22.47 | | 95.32 | 21.69 | | 3.64^**^ |
| Picture Sequence Memory Test | | 100.74 | 16.16 | 100.09 | 16.68 | | 101.28 | 15.46 | | 101.04 | 16.20 | | 102.57 | 16.76 | | 2.60^*^ |
| Oral Reading Recognition Test | | 102.37 | 19.01 | 102.65 | 20.48 | | 104.32 | 20.44 | | 100.24 | 15.26 | | 103.49 | 18.43 | | 2.51 |
| Crystallized Composite | | 106.33 | 19.05 | 106.96 | 20.97 | | 107.59 | 19.74 | | 104.38 | 15.54 | | 106.93 | 17.55 | | 1.59 |
|  | | N | % | N | % | | N | % | | N | % | | N | % | |  |
| Female | | 388 | 49.4% | 153 | 49.68% | | 113 | 49.34% | | 125 | 50.20% | | 1561 | 45.18% | | 1.54 |
| Grandparents’ psychiatric history | | 99 | 12.6% | 27 | 8.77% | | 30 | 13.10% | | 45 | 18.07% | | 614 | 17.77% | | 70.19^***^ |
| Parents’ psychiatric history | | 138 | 17.6% | 56 | 18.18% | | 40 | 17.47% | | 45 | 18.07% | | 518 | 14.99% | |  |
| Grandparents and parents’ psychiatric history | | 402 | 51.1% | 191 | 62.01% | | 112 | 48.91% | | 102 | 40.96% | | 1012 | 29.29% | |  |
| Left-handedness | | 47 | 6.0% | 21 | 6.82% | | 14 | 6.11% | | 15 | 6.02% | | 229 | 6.63% | | 1.28 |
| Mixed-handedness | | 123 | 15.6% | 45 | 14.61% | | 43 | 18.78% | | 38 | 15.26% | | 460 | 13.31% | |  |
| Race (white) | | 650 | 82.7% | 263 | 85.4% | | 191 | 83.4% | | 196 | 78.7% | | 2690 | 77.9% | | 2.37 |
| Race (black) | | 76 | 9.7% | 26 | 8.4% | | 20 | 8.7% | | 30 | 12.0% | | 466 | 13.5% | |  |
| Race (American Indian) | | 6 | 0.8% | 1 | 0.3% | | 2 | 0.9% | | 3 | 1.2% | | 23 | 0.7% | |  |
| Race (other) | | 54 | 6.9% | 18 | 5.8% | | 16 | 7.0% | | 20 | 8.0% | | 276 | 8.0% | |  |
| **Mental health** | |  | |  | | |  | | |  | | |  | | |  |
|  | | Mean | S.D. | Mean | | S.D. | Mean | | S.D. | Mean | | S.D. | Mean | | S.D. |  |
| Prodromal Psychosis | | 8.18 | 12.86 | 9.38 | 14.51 | | 8.60 | 12.70 | | 6.31 | 10.44 | | 5.53 | 9.71 | | 18.23^***^ |
| Mean severity of Prodromal Psychosis | | 2.34 | 1.17 | 2.40 | 1.18 | | 2.25 | 1.10 | | 2.34 | 1.22 | | 2.02 | 1.05 | | 12.56^***^ |
| Lack of Premeditation | | 7.66 | 2.48 | 7.64 | 2.50 | | 7.67 | 2.34 | | 7.66 | 2.59 | | 7.67 | 2.30 | | 0.02 |
| Lack of Perseverance | | 7.30 | 2.46 | 7.62 | 2.57 | | 7.09 | 2.42 | | 7.09 | 2.32 | | 6.84 | 2.09 | | 13.22^***^ |
| Negative Urgency | | 8.65 | 2.75 | 8.63 | 2.80 | | 8.79 | 2.69 | | 8.56 | 2.76 | | 8.28 | 2.58 | | 4.70^**^ |
| Sensation Seeking | | 9.54 | 2.82 | 9.53 | 2.85 | | 9.42 | 2.82 | | 9.67 | 2.79 | | 9.95 | 2.62 | | 5.39^**^ |
| Behavioral Inhibition | | 10.12 | 3.88 | 10.29 | 3.88 | | 10.20 | 3.69 | | 9.83 | 4.05 | | 9.04 | 3.62 | | 19.38^***^ |
| Depression | | 58.58 | 7.35 | 61.19 | 7.82 | | 57.74 | 6.81 | | 56.12 | 6.13 | | 51.53 | 3.19 | | 681.22^***^ |
| Somatic | | 59.39 | 7.63 | 60.15 | 8.09 | | 60.00 | 6.88 | | 57.90 | 7.52 | | 53.99 | 5.48 | | 185.36^***^ |
| ADHD | | 56.59 | 7.34 | 58.60 | 8.14 | | 56.00 | 7.01 | | 54.63 | 5.83 | | 51.73 | 3.89 | | 266.29^***^ |
| Sluggish Cognitive Tempo | | 55.98 | 7.04 | 57.73 | 7.70 | | 55.46 | 6.30 | | 54.28 | 6.35 | | 51.68 | 3.82 | | 218.97^***^ |
| Oppositional defiance | | 57.00 | 7.12 | 58.51 | 7.90 | | 56.12 | 6.35 | | 55.93 | 6.46 | | 52.05 | 3.71 | | 275.39^***^ |
| Conduct disorder | | 55.30 | 6.86 | 56.22 | 7.51 | | 54.47 | 5.84 | | 54.91 | 6.79 | | 51.70 | 3.82 | | 142.05^***^ |
| **Stress indicators** | |  | |  | | |  | | |  | | |  | | |  |
| **Demographics** | |  | |  | | |  | | |  | | |  | | |  |
| Parents Education | | 16.58 | 2.68 | 16.62 | 2.54 | | 16.72 | 2.72 | | 16.41 | 2.82 | | 17.01 | 2.53 | | 6.50^***^ |
| Family income | | 7.14 | 2.28 | 6.94 | 2.37 | | 7.26 | 2.12 | | 7.27 | 2.32 | | 7.61 | 2.22 | | 10.23^***^ |
| Area Deprivation Index | | 40.07 | 25.72 | 38.55 | 25.18 | | 39.18 | 25.28 | | 42.79 | 26.68 | | 37.05 | 25.42 | | 4.16^**^ |
| Body mass index | | 18.96 | 4.38 | 19.02 | 4.62 | | 19.33 | 4.61 | | 18.53 | 3.81 | | 18.50 | 3.87 | | 4.29^**^ |
|  | | N | % | N | % | | N | % | | N | % | | N | % | |  |
| Recent social deprivation | | 203 | 25.8% | 91 | 29.55% | | 57 | 24.89% | | 58 | 23.29% | | 527 | 15.25% | | 19.04^***^ |
| **Physical health** | |  | |  | | |  | | |  | | |  | | |  |
|  | | Mean | S.D. | Mean | S.D. | | Mean | S.D. | | Mean | S.D. | | Mean | S.D. | |  |
| Initiating and maintaining sleep disorders | | 13.89 | 4.26 | 14.86 | 4.58 | | 13.87 | 4.03 | | 12.69 | 3.73 | | 10.63 | 2.87 | | 249.96^***^ |
| Sleep breathing disorders | | 3.97 | 1.35 | 4.02 | 1.34 | | 4.00 | 1.47 | | 3.86 | 1.23 | | 3.63 | 1.08 | | 19.83^***^ |
| Arousal or nightmares disorders | | 3.78 | 1.18 | 3.88 | 1.22 | | 3.82 | 1.27 | | 3.63 | 1.01 | | 3.30 | 0.70 | | 79.23^***^ |
| Sleep wake transition disorders | | 9.55 | 3.27 | 10.36 | 3.55 | | 9.45 | 3.29 | | 8.65 | 2.58 | | 7.62 | 2.10 | | 170.13^***^ |
| Excessive somnolence disorders | | 8.09 | 3.00 | 8.69 | 3.27 | | 7.93 | 3.02 | | 7.49 | 2.47 | | 6.39 | 1.86 | | 154.02^***^ |
| Sleep hyperhidrosis | | 2.73 | 1.61 | 2.96 | 1.90 | | 2.71 | 1.53 | | 2.47 | 1.19 | | 2.27 | 0.87 | | 52.50^***^ |
| Screen time during weekdays | | 3.54 | 3.12 | 3.66 | 3.15 | | 3.60 | 3.20 | | 3.33 | 3.01 | | 3.16 | 2.87 | | 4.20^**^ |
| Physical activity | | 3.45 | 2.27 | 3.37 | 2.32 | | 3.26 | 2.26 | | 3.74 | 2.21 | | 3.67 | 2.29 | | 3.98^**^ |
|  | | N | % | N | % | | N | % | | N | % | | N | % | |  |
| Substance use | | 187 | 23.8% | 73 | 23.70% | | 53 | 23.14% | | 64 | 25.70% | | 854 | 24.72% | | 0.25 |
| **Environment** | |  | |  | | |  | | |  | | |  | | |  |
| Family | |  | |  | | |  | | |  | | |  | | |  |
|  | | Mean | S.D. | Mean | S.D. | | Mean | S.D. | | Mean | S.D. | | Mean | S.D. | |  |
| Multi-group ethnic identity (caregiver) | | 3.36 | 0.88 | 3.29 | 0.91 | | 3.42 | 0.80 | | 3.40 | 0.93 | | 3.33 | 0.87 | | 1.23 |
| Neighborhood safety | | 3.88 | 0.98 | 3.87 | 0.99 | | 3.77 | 0.97 | | 3.98 | 0.98 | | 4.01 | 0.91 | | 6.85^***^ |
| Family conflict | | 2.10 | 2.00 | 2.13 | 2.04 | | 1.93 | 1.87 | | 2.20 | 2.07 | | 1.90 | 1.88 | | 3.03^*^ |
| Acceptance by parent | | 2.78 | 0.29 | 2.79 | 0.28 | | 2.79 | 0.28 | | 2.76 | 0.31 | | 2.79 | 0.29 | | 0.60 |
| Prosocial behavior (child report) | | 1.67 | 0.37 | 1.68 | 0.36 | | 1.65 | 0.38 | | 1.66 | 0.38 | | 1.68 | 0.36 | | 0.40 |
| Prosocial behavior (parent report) | | 1.71 | 0.40 | 1.70 | 0.40 | | 1.67 | 0.43 | | 1.74 | 0.37 | | 1.78 | 0.38 | | 10.48^***^ |
| School | |  | |  | | |  | | |  | | |  | | |  |
| School environment | | 19.62 | 2.95 | 19.53 | 3.03 | | 19.49 | 2.77 | | 19.84 | 3.02 | | 20.14 | 2.61 | | 9.06^***^ |
| School disengagement | | 3.84 | 1.53 | 3.86 | 1.53 | | 3.83 | 1.48 | | 3.83 | 1.59 | | 3.65 | 1.40 | | 3.81^*^ |
| Friends(girls) | | 3.57 | 7.71 | 3.77 | 8.54 | | 3.86 | 9.41 | | 3.06 | 3.95 | | 3.19 | 5.58 | | 1.71 |
| Friends(boys) | | 3.14 | 6.42 | 2.76 | 4.01 | | 4.12 | 10.20 | | 2.71 | 3.64 | | 3.09 | 4.87 | | 3.85^**^ |
| **Mental health** | |  | |  | | |  | | |  | | |  | | |  |
| Traumatic history | | 0.66 | 0.93 | 0.77 | 1.04 | | 0.63 | 0.88 | | 0.54 | 0.81 | | 0.42 | 0.95 | | 15.61^***^ |

^***^, *p*<0.001

^**^, *p*<0.01

^*^, *p*<0.05

##

## Table S**4** Differences in baseline variables for eligible participants and excluded subthreshold anxiety groups (N=2421).

|  | Eligible  (N=786) | | Excluded  (N=1635) | | | *t* | |  |
| --- | --- | --- | --- | --- | --- | --- | --- | --- |
| **Diathesis indicators** |  |  |  |  |  |  |  |  |
| **Demographics** |  | |  | | |  | |  |
|  | Mean | S.D. | | Mean | S.D. | |  | |
| Age | 118.95 | 7.65 | | 118.82 | 7.56 | | 0.41 | |
|  | N | % | | N | % | |  | |
| Female | 388 | 49.4 | | 773 | 47.3 | | 0.96 | |
| Grandparents’ psychiatric history | 99 | 12.6 | | 198 | 12.1 | | 1.84 | |
| Parents’ psychiatric history | 138 | 17.6 | | 271 | 16.6 | |  |  |
| Grandparents and parents’ psychiatric history | 402 | 51.1 | | 793 | 48.5 | |  |  |
| Left-handedness | 47 | 6.0 | | 161 | 9.8 | | 0.13 | |
| Mixed-handedness | 123 | 15.6 | | 214 | 13.1 | |  |  |
| **Mental health** |  | |  | | |  | |  |
|  | Mean | S.D. | | Mean | S.D. | |  | |
| Prodromal Psychosis | 8.18 | 12.86 | | 7.54 | 11.57 | | 1.22 | |
| Mean severity of Prodromal Psychosis | 2.34 | 1.17 | | 2.24 | 1.11 | | 1.54 | |
| Lack of Premeditation | 7.66 | 2.48 | | 7.8 | 2.59 | | -1.34 | |
| Lack of Perseverance | 7.3 | 2.46 | | 7.25 | 2.43 | | 0.45 | |
| Negative Urgency | 8.65 | 2.75 | | 8.62 | 2.90 | | 0.25 | |
| Sensation Seeking | 9.54 | 2.82 | | 9.46 | 2.85 | | 0.67 | |
| Behavioral Inhibition | 10.12 | 3.88 | | 9.93 | 3.93 | | 1.08 | |
| Depression | 58.58 | 7.35 | | 58.14 | 6.97 | | 1.42 | |
| **Somatic** | **59.39** | **7.63** | | **58.68** | **7.61** | | **2.15^*^** | |
| ADHD | 56.59 | 7.34 | | 56.57 | 7.24 | | 0.04 | |
| Sluggish Cognitive Tempo | 55.98 | 7.04 | | 56.08 | 7.04 | | -0.34 | |
| Oppositional defiance | 57 | 7.12 | | 56.6 | 6.91 | | 1.31 | |
| Conduct disorder | 55.3 | 6.86 | | 55.89 | 7.44 | | -1.93 | |
| **Stress indicators** |  | |  | | |  | |  |
| **Demographics** |  | |  | | |  | |  |
| Parents Education | 3.54 | 1.12 | | 3.49 | 1.16 | | 1.05 | |
| Family income | 2.09 | 0.83 | | 2.02 | 0.84 | | 1.62 | |
| Body mass index | 18.96 | 4.38 | | 18.88 | 4.39 | | 0.37 | |
| Area Deprivation Index | 40.07 | 25.72 | | 41.5 | 27.49 | | -1.21 | |
|  | N | % | | N | % | |  | |
| Recent social deprivation | 203 | 25.8 | | 479 | 29.3 | | -1.91 | |
| **Physical health** |  | |  | | |  | |  |
|  | Mean | S.D. | | Mean | S.D. | |  | |
| Initiating and maintaining sleep disorders | 13.89 | 4.26 | | 13.77 | 4.34 | | 0.60 | |
| Sleep breathing disorders | 3.97 | 1.35 | | 3.96 | 1.41 | | 0.05 | |
| Disorders of arousal or nightmares | 3.78 | 1.18 | | 3.73 | 1.15 | | 1.14 | |
| Sleep wake transition disorders | 9.55 | 3.27 | | 9.34 | 3.09 | | 1.56 | |
| Excessive somnolence disorders | 8.09 | 3.00 | | 8.03 | 2.90 | | 0.42 | |
| Sleep hyperhidrosis | 2.73 | 1.61 | | 2.76 | 1.54 | | -0.39 | |
| Screen time during weekdays | 3.54 | 3.12 | | 3.68 | 3.18 | | -1.02 | |
| Physical activity | 3.45 | 2.27 | | 3.29 | 2.31 | | 1.65 | |
|  | N | % | | N | % | |  | |
| Substance use | 187 | 23.8 | | 387 | 23.7 | | 0.07 | |
| **Environment** |  | |  | | |  | |  |
| Family |  | |  | | |  | |  |
|  | Mean | S.D. | | Mean | S.D. | |  | |
| Multi-group ethnic identity (caregiver) | 3.36 | 0.89 | | 3.32 | 0.89 | | 0.96 | |
| **Neighborhood safety** | **3.88** | **0.98** | | **3.77** | **0.99** | | **2.51^*^** | |
| Family conflict | 2.1 | 2.00 | | 2.2 | 2.01 | | -1.24 | |
| Prosocial behavior (child report) | 1.67 | 0.37 | | 1.66 | 0.38 | | 0.46 | |
| Prosocial behavior (parent report) | 1.71 | 0.40 | | 1.68 | 0.44 | | 1.70 | |
| Acceptance by parent | 2.78 | 0.29 | | 2.76 | 0.32 | | 1.40 | |
| School |  | |  | | |  | |  |
| School environment | 19.62 | 2.95 | | 19.69 | 3.04 | | -0.52 | |
| School disengagement | 3.84 | 1.54 | | 3.82 | 1.51 | | 0.34 | |
| Friends(girls) | 3.57 | 7.71 | | 3.28 | 6.50 | | 0.98 | |
| Friends(boys) | 3.14 | 6.42 | | 2.8 | 5.15 | | 1.30 | |
| **Mental health** |  | |  | | |  | |  |
| Traumatic history | 0.66 | 0.93 | | 0.7 | 1.03 | | -1.04 | |

^***^, *p*<0.001

^**^, *p*<0.01

^*^, *p*<0.05

## Table S**5** Multinomial logistic regression analysis (STA→HCs, STA→STA, STA→AX, and HCs).

|  |  | Odds ratio | 95%CIs | | *β* | *z* | *Wald statistic* | *p* |
| --- | --- | --- | --- | --- | --- | --- | --- | --- |
|  |  |  | Lower | Upper |  |  |  |  |
| **Comparison** | **Variables** |  |  |  |  |  |  |  |
| **STA→HCs vs HCs** | **Diathesis indicators** |  |  |  |  |  |  |  |
|  | **Demographics** |  |  |  |  |  |  |  |
|  | **Age** | **0.74** | **0.64** | **0.85** | **-0.31** | **-4.12** | **16.99^**^** | **<0.001** |
|  | Female | 1.31 | 0.94 | 1.85 | 0.27 | 1.58 | 2.49 | 0.11 |
|  | Grandparents’ psychiatric history | 1.34 | 0.86 | 2.03 | 0.29 | 1.33 | 1.77 | 0.18 |
|  | Parents’ psychiatric history | 1.4 | 0.88 | 2.08 | 0.34 | 1.53 | 2.35 | 0.13 |
|  | Grandparents and parents’ psychiatric history | 1.3 | 0.90 | 1.85 | 0.26 | 1.41 | 2 | 0.16 |
|  | Left-handedness | 0.88 | 0.48 | 1.59 | -0.12 | -0.41 | 0.17 | 0.68 |
|  | Mixed-handedness | 1.03 | 0.70 | 1.54 | 0.03 | 0.14 | 0.02 | 0.89 |
|  | **Mental health** |  |  |  |  |  |  |  |
|  | Prodromal Psychosis | 0.79 | 0.65 | 0.96 | -0.24 | -2.44 | 5.94 | <0.05 |
|  | Mean severity of Prodromal Psychosis | 1.23 | 1.03 | 1.43 | 0.21 | 2.57 | 6.62 | <0.05 |
|  | Lack of Premeditation | 0.91 | 0.78 | 1.08 | -0.09 | -1.1 | 1.22 | 0.27 |
|  | Lack of Perseverance | 1 | 0.85 | 1.17 | 0 | -0.06 | 0 | 0.95 |
|  | Negative Urgency | 0.99 | 0.85 | 1.15 | -0.01 | -0.17 | 0.03 | 0.86 |
|  | Sensation Seeking | 0.91 | 0.79 | 1.05 | -0.1 | -1.34 | 1.78 | 0.18 |
|  | Behavioral Inhibition | 1.19 | 1.03 | 1.38 | 0.18 | 2.36 | 5.58 | <0.05 |
|  | **Depression** | **1.87** | **1.60** | **2.20** | **0.62** | **7.6** | **57.73^***^** | **<0.001** |
|  | **Somatic** | **1.43** | **0.85** | **1.18** | **0.36** | **5.25** | **27.53^***^** | **<0.001** |
|  | ADHD | 1 | 1.25 | 1.63 | 0 | 0.06 | 0 | 0.95 |
|  | Sluggish Cognitive Tempo | 1.18 | 1.02 | 1.35 | 0.16 | 2.25 | 5.07 | <0.05 |
|  | **Oppositional defiance** | **1.42** | **1.19** | **1.69** | **0.35** | **3.95** | **15.57^**^** | **<0.001** |
|  | Conduct disorder | 1.05 | 0.89 | 1.24 | 0.05 | 0.61 | 0.37 | 0.54 |
|  | **Stress indicators** |  |  |  |  |  |  |  |
|  | **Demographics** |  |  |  |  |  |  |  |
|  | Parents Education | 0.8 | 0.66 | 0.94 | -0.22 | -2.55 | 6.52 | <0.05 |
|  | Family income | 1.24 | 1.06 | 1.57 | 0.21 | 2.14 | 4.56 | <0.05 |
|  | Recent social deprivation | 1.07 | 0.72 | 1.59 | 0.06 | 0.31 | 0.1 | 0.75 |
|  | Area Deprivation Index | 1.18 | 1.03 | 1.42 | 0.16 | 1.97 | 3.88 | <0.05 |
|  | Body mass index | 0.99 | 0.85 | 1.14 | -0.01 | -0.1 | 0.01 | 0.92 |
|  | **Physical health** |  |  |  |  |  |  |  |
|  | Initiating and maintaining sleep disorders | 1.24 | 1.07 | 1.45 | 0.22 | 2.8 | 7.86 | <0.05 |
|  | Sleep breathing disorders | 0.93 | 0.82 | 1.08 | -0.07 | -0.94 | 0.89 | 0.35 |
|  | Disorders of arousal or nightmares | 1.19 | 1.05 | 1.35 | 0.17 | 2.72 | 7.39 | <0.05 |
|  | Sleep wake transition disorders | 1.09 | 0.93 | 1.26 | 0.09 | 1.11 | 1.24 | 0.26 |
|  | Excessive somnolence disorders | 0.99 | 0.85 | 1.15 | -0.01 | -0.15 | 0.02 | 0.88 |
|  | Sleep hyperhidrosis | 0.97 | 0.85 | 1.11 | -0.03 | -0.39 | 0.16 | 0.69 |
|  | Screen time during weekdays | 0.96 | 0.83 | 1.11 | -0.04 | -0.52 | 0.27 | 0.60 |
|  | Physical activity | 1.13 | 0.98 | 1.31 | 0.13 | 1.71 | 2.93 | 0.09 |
|  | Substance use | 1.06 | 0.76 | 1.47 | 0.06 | 0.35 | 0.12 | 0.72 |
|  | **Environment** |  |  |  |  |  |  |  |
|  | Family |  |  |  |  |  |  |  |
|  | Multi-group ethnic identity (caregiver) | 1.12 | 1.00 | 1.33 | 0.11 | 1.51 | 2.27 | 0.13 |
|  | Neighborhood safety | 1.2 | 1.02 | 1.39 | 0.18 | 2.29 | 5.25 | <0.05 |
|  | Family conflict | 0.92 | 0.79 | 1.07 | -0.09 | -1.13 | 1.27 | 0.26 |
|  | Prosocial behavior (child report) | 0.97 | 0.99 | 1.36 | -0.03 | -0.39 | 0.15 | 0.70 |
|  | Prosocial behavior (parent report) | 1.17 | 0.83 | 1.15 | 0.15 | 1.9 | 3.61 | 0.06 |
|  | Acceptance by parent | 0.97 | 0.84 | 1.12 | -0.03 | -0.38 | 0.14 | 0.71 |
|  | School |  |  |  |  |  |  |  |
|  | School environment | 0.94 | 0.80 | 1.08 | -0.06 | -0.8 | 0.64 | 0.42 |
|  | School disengagement | 0.99 | 0.85 | 1.14 | -0.01 | -0.11 | 0.01 | 0.91 |
|  | Friends(girls) | 0.91 | 0.75 | 1.09 | -0.1 | -1.03 | 1.07 | 0.30 |
|  | Friends(boys) | 0.93 | 0.76 | 1.14 | -0.07 | -0.73 | 0.54 | 0.46 |
|  | **Mental health** |  |  |  |  |  |  |  |
|  | Traumatic history | 0.99 | 0.85 | 1.15 | -0.01 | -0.17 | 0.03 | 0.86 |
| **STA→STA vs HCs** | **Diathesis indicators** |  |  |  |  |  |  |  |
|  | **Demographics** |  |  |  |  |  |  |  |
|  | Age | 1.08 | 0.92 | 1.26 | 0.08 | 0.97 | 0.94 | 0.33 |
|  | Female | 1.54 | 1.09 | 2.19 | 0.43 | 2.43 | 5.91 | <0.05 |
|  | Grandparents’ psychiatric history | 1.15 | 0.73 | 2.05 | 0.14 | 0.52 | 0.27 | 0.61 |
|  | Parents’ psychiatric history | 1.43 | 0.87 | 2.32 | 0.35 | 1.42 | 2.02 | 0.15 |
|  | Grandparents and parents’ psychiatric history | 1.72 | 1.18 | 2.62 | 0.54 | 2.68 | 7.21 | <0.01 |
|  | Left-handedness | 0.94 | 0.49 | 1.79 | -0.07 | -0.2 | 0.04 | 0.84 |
|  | Mixed-handedness | 1.27 | 0.85 | 1.93 | 0.24 | 1.14 | 1.3 | 0.25 |
|  | **Mental health** |  |  |  |  |  |  |  |
|  | Prodromal Psychosis | 1.05 | 0.88 | 1.26 | 0.05 | 0.51 | 0.26 | 0.61 |
|  | Mean severity of Prodromal Psychosis | 1.05 | 0.86 | 1.25 | 0.05 | 0.54 | 0.29 | 0.59 |
|  | Lack of Premeditation | 0.86 | 0.72 | 1.04 | -0.15 | -1.6 | 2.56 | 0.11 |
|  | Lack of Perseverance | 0.89 | 0.74 | 1.05 | -0.12 | -1.33 | 1.78 | 0.18 |
|  | Negative Urgency | 1.12 | 0.95 | 1.32 | 0.12 | 1.38 | 1.9 | 0.17 |
|  | **Sensation Seeking** | **0.76** | **0.64** | **0.88** | **-0.28** | **-3.48** | **12.08^*^** | **<0.01** |
|  | Behavioral Inhibition | 1.26 | 1.08 | 1.47 | 0.23 | 2.85 | 8.14 | <0.01 |
|  | **Depression** | **1.97** | **1.67** | **2.31** | **0.68** | **8.22** | **67.56^***^** | **<0.001** |
|  | **Somatic** | **1.66** | **1.00** | **1.37** | **0.5** | **6.98** | **48.71^***^** | **<0.001** |
|  | ADHD | 1.17 | 1.44 | 1.91 | 0.16 | 1.93 | 3.71 | 0.05 |
|  | Sluggish Cognitive Tempo | 1.19 | 1.03 | 1.37 | 0.17 | 2.39 | 5.7 | <0.05 |
|  | Oppositional defiance | 1.35 | 1.12 | 1.64 | 0.3 | 3.17 | 10.02 | <0.01 |
|  | Conduct disorder | 0.82 | 0.68 | 0.98 | -0.2 | -2.22 | 4.91 | <0.05 |
|  | **Stress indicators** |  |  |  |  |  |  |  |
|  | **Demographics** |  |  |  |  |  |  |  |
|  | Parents Education | 1.06 | 0.88 | 1.29 | 0.06 | 0.62 | 0.38 | 0.54 |
|  | Family income | 1 | 0.77 | 1.17 | 0 | -0.03 | 0 | 0.98 |
|  | Recent social deprivation | 1.04 | 0.65 | 1.51 | 0.04 | 0.18 | 0.03 | 0.85 |
|  | Area Deprivation Index | 0.91 | 0.72 | 1.04 | -0.09 | -1.01 | 1.02 | 0.31 |
|  | Body mass index | 1.08 | 0.95 | 1.27 | 0.08 | 1.08 | 1.16 | 0.28 |
|  | **Physical health** |  |  |  |  |  |  |  |
|  | **Initiating and maintaining sleep disorders** | **1.43** | **1.23** | **1.67** | **0.36** | **4.56** | **20.78^***^** | **<0.001** |
|  | Sleep breathing disorders | 0.98 | 0.85 | 1.13 | -0.02 | -0.3 | 0.09 | 0.76 |
|  | Disorders of arousal or nightmares | 1.23 | 1.08 | 1.39 | 0.2 | 3.15 | 9.93 | <0.01 |
|  | Sleep wake transition disorders | 1.24 | 1.06 | 1.43 | 0.21 | 2.77 | 7.65 | <0.01 |
|  | Excessive somnolence disorders | 0.92 | 0.79 | 1.08 | -0.08 | -1.06 | 1.12 | 0.29 |
|  | Sleep hyperhidrosis | 1.04 | 0.92 | 1.19 | 0.04 | 0.66 | 0.44 | 0.51 |
|  | Screen time during weekdays | 0.98 | 0.84 | 1.15 | -0.02 | -0.29 | 0.08 | 0.77 |
|  | Physical activity | 0.86 | 0.73 | 1.01 | -0.15 | -1.85 | 3.42 | 0.06 |
|  | Substance use | 0.87 | 0.60 | 1.26 | -0.13 | -0.71 | 0.51 | 0.48 |
|  | **Environment** |  |  |  |  |  |  |  |
|  | Family |  |  |  |  |  |  |  |
|  | Multi-group ethnic identity (caregiver) | 1.17 | 1.00 | 1.37 | 0.16 | 2.01 | 4.03 | <0.05 |
|  | Neighborhood safety | 0.96 | 0.82 | 1.12 | -0.04 | -0.47 | 0.22 | 0.64 |
|  | Family conflict | 0.79 | 0.67 | 0.94 | -0.23 | -2.68 | 7.2 | <0.01 |
|  | Prosocial behavior (child report) | 0.88 | 0.78 | 1.06 | -0.13 | -1.45 | 2.11 | 0.15 |
|  | Prosocial behavior (parent report) | 0.91 | 0.74 | 1.05 | -0.09 | -1.12 | 1.26 | 0.26 |
|  | Acceptance by parent | 1.13 | 0.95 | 1.34 | 0.13 | 1.44 | 2.09 | 0.15 |
|  | School |  |  |  |  |  |  |  |
|  | School environment | 0.79 | 0.68 | 0.93 | -0.23 | -2.84 | 8.05 | <0.01 |
|  | School disengagement | 0.95 | 0.80 | 1.12 | -0.06 | -0.67 | 0.45 | 0.50 |
|  | Friends(girls) | 1.01 | 0.88 | 1.15 | 0.01 | 0.15 | 0.02 | 0.88 |
|  | Friends(boys) | 1.17 | 1.04 | 1.31 | 0.16 | 2.63 | 6.93 | <0.01 |
|  | **Mental health** |  |  |  |  |  |  |  |
|  | Traumatic history | 1.03 | 0.92 | 1.21 | 0.03 | 0.43 | 0.19 | 0.67 |
| **STA→AX vs HCs** | **Diathesis indicators** |  |  |  |  |  |  |  |
|  | **Demographics** |  |  |  |  |  |  |  |
|  | Age | 0.97 | 0.82 | 1.12 | -0.04 | -0.45 | 0.2 | 0.66 |
|  | Female | 1.35 | 0.94 | 1.93 | 0.3 | 1.64 | 2.68 | 0.10 |
|  | Grandparents’ psychiatric history | 1.26 | 0.71 | 2.39 | 0.23 | 0.75 | 0.56 | 0.45 |
|  | **Parents’ psychiatric history** | **2.76** | **1.61** | **4.61** | **1.02** | **3.8** | **14.41^***^** | **<0.001** |
|  | **Grandparents and parents’ psychiatric history** | **3.33** | **2.16** | **5.27** | **1.2** | **5.29** | **28.03^***^** | **<0.001** |
|  | Left-handedness | 1.16 | 0.63 | 2.10 | 0.15 | 0.49 | 0.24 | 0.62 |
|  | Mixed-handedness | 0.93 | 0.60 | 1.43 | -0.08 | -0.35 | 0.12 | 0.73 |
|  | **Mental health** |  |  |  |  |  |  |  |
|  | Prodromal Psychosis | 1.07 | 0.90 | 1.28 | 0.07 | 0.78 | 0.61 | 0.43 |
|  | Mean severity of Prodromal Psychosis | 1.09 | 0.90 | 1.30 | 0.09 | 0.95 | 0.9 | 0.34 |
|  | **Lack of Premeditation** | **0.74** | **0.61** | **0.88** | **-0.31** | **-3.29** | **10.82^*^** | **<0.01** |
|  | Lack of Perseverance | 1.15 | 0.96 | 1.34 | 0.14 | 1.56 | 2.43 | 0.12 |
|  | Negative Urgency | 0.99 | 0.84 | 1.16 | -0.01 | -0.12 | 0.01 | 0.91 |
|  | Sensation Seeking | 0.79 | 0.68 | 0.92 | -0.23 | -2.9 | 8.41 | <0.01 |
|  | Behavioral Inhibition | 1.27 | 1.09 | 1.49 | 0.24 | 2.98 | 8.89 | <0.01 |
|  | **Depression** | **2.54** | **2.17** | **2.95** | **0.93** | **11.83** | **139.99^***^** | **<0.001** |
|  | **Somatic** | **1.38** | **1.04** | **1.40** | **0.32** | **4.61** | **21.26^***^** | **<0.001** |
|  | ADHD | 1.2 | 1.21 | 1.59 | 0.18 | 2.41 | 5.8 | 0.02 |
|  | **Sluggish Cognitive Tempo** | **1.33** | **1.17** | **1.53** | **0.29** | **4.31** | **18.57^***^** | **<0.001** |
|  | **Oppositional defiance** | **1.47** | **1.24** | **1.76** | **0.39** | **4.28** | **18.33^***^** | **<0.001** |
|  | Conduct disorder | 0.86 | 0.72 | 1.02 | -0.16 | -1.79 | 3.2 | 0.07 |
|  | **Stress indicators** |  |  |  |  |  |  |  |
|  | **Demographics** |  |  |  |  |  |  |  |
|  | Parents Education | 0.99 | 0.82 | 1.20 | -0.01 | -0.07 | 0 | 0.95 |
|  | Family income | 0.89 | 0.71 | 1.07 | -0.11 | -1.09 | 1.18 | 0.28 |
|  | Body mass index | 1.03 | 0.92 | 1.24 | 0.03 | 0.42 | 0.18 | 0.67 |
|  | Recent social deprivation | 0.97 | 0.60 | 1.37 | -0.03 | -0.14 | 0.02 | 0.88 |
|  | **Area Deprivation Index** | **1.32** | **0.67** | **0.97** | **0.28** | **0.99** | **12.65^*^** | **<0.01** |
|  | **Physical health** |  |  |  |  |  |  |  |
|  | **Initiating and maintaining sleep disorders** | **1.46** | **1.26** | **1.70** | **0.38** | **4.95** | **24.54^***^** | **<0.001** |
|  | Sleep breathing disorders | 0.89 | 0.76 | 1.03 | -0.12 | -1.53 | 2.33 | 0.13 |
|  | Disorders of arousal or nightmares | 1.12 | 0.99 | 1.28 | 0.12 | 1.77 | 3.14 | 0.08 |
|  | **Sleep wake transition disorders** | **1.47** | **1.27** | **1.69** | **0.39** | **5.33** | **28.38^***^** | **<0.001** |
|  | Excessive somnolence disorders | 0.89 | 0.77 | 1.04 | -0.11 | -1.47 | 2.17 | 0.14 |
|  | Sleep hyperhidrosis | 1.09 | 0.96 | 1.23 | 0.09 | 1.4 | 1.96 | 0.16 |
|  | Screen time during weekdays | 0.96 | 0.82 | 1.13 | -0.04 | -0.49 | 0.24 | 0.62 |
|  | Physical activity | 1 | 0.85 | 1.17 | 0 | -0.01 | 0 | 1.00 |
|  | Substance use | 0.97 | 0.68 | 1.39 | -0.04 | -0.19 | 0.04 | 0.85 |
|  | **Environment** |  |  |  |  |  |  |  |
|  | Family |  |  |  |  |  |  |  |
|  | Multi-group ethnic identity (caregiver) | 1.07 | 0.91 | 1.24 | 0.06 | 0.83 | 0.69 | 0.41 |
|  | Neighborhood safety | 1.13 | 0.97 | 1.32 | 0.12 | 1.5 | 2.25 | 0.13 |
|  | Family conflict | 0.84 | 0.72 | 1.00 | -0.17 | -2.05 | 4.2 | <0.05 |
|  | Prosocial behavior (child report) | 0.99 | 0.97 | 1.35 | -0.01 | -0.15 | 0.02 | 0.88 |
|  | Prosocial behavior (parent report) | 1.14 | 0.83 | 1.18 | 0.13 | 1.59 | 2.51 | 0.11 |
|  | Acceptance by parent | 1.07 | 0.91 | 1.27 | 0.07 | 0.82 | 0.67 | 0.41 |
|  | School |  |  |  |  |  |  |  |
|  | School environment | 0.85 | 0.72 | 0.99 | -0.16 | -1.97 | 3.88 | <0.05 |
|  | School disengagement | 0.92 | 0.77 | 1.08 | -0.09 | -1.05 | 1.11 | 0.29 |
|  | Friends(girls) | 1.05 | 0.91 | 1.20 | 0.05 | 0.68 | 0.46 | 0.50 |
|  | Friends(boys) | 0.91 | 0.74 | 1.11 | -0.1 | -0.93 | 0.86 | 0.35 |
|  | **Mental health** |  |  |  |  |  |  |  |
|  | Traumatic history | 1.06 | 0.94 | 1.22 | 0.06 | 0.92 | 0.85 | 0.36 |

^***^, *p*<0.001 following Bonferroni correction.

^**^, *p*<0.01 following Bonferroni correction.

^*^, *p*<0.05 following Bonferroni correction.

## Table S**6** Differences in variables for baseline subthreshold anxiety and healthy controls (N=11556).

|  | Subthreshold anxiety (N=2266) | | Healthy controls (N=9290) | | *t* |
| --- | --- | --- | --- | --- | --- |
| **Diathesis indicators** |  |  |  |  |  |
| **Demographics** |  | |  | |  |
|  | Mean | S.D. | Mean | S.D. |  |
| Age | 118.79 | 7.58 | 119.02 | 7.47 | -1.31 |
|  | N | % | N | % |  |
| Female | 1076 | 47.5 | 4458 | 48.0 | -0.43 |
| **Grandparents’ psychiatric history** | **284** | **12.5** | **1539** | **16.6** | **16.28^***^** |
| **Parents’ psychiatric history** | **389** | **17.2** | **1468** | **15.8** |  |
| **Grandparents and parents’ psychiatric history** | **1096** | **48.4** | **3027** | **32.6** |  |
| **Left-handedness** | **200** | **8.8** | **627** | **6.7** | **2.13^*^** |
| **Mixed-handedness** | **319** | **14.1** | **1239** | **13.3** |  |
| **Mental health** |  | |  | |  |
|  | Mean | S.D. | Mean | S.D. |  |
| **Prodromal Psychosis** | **7.61** | **11.75** | **5.93** | **10.15** | **6.25^***^** |
| **Mean severity of Prodromal Psychosis** | **2.26** | **1.13** | **2.11** | **1.08** | **4.73^***^** |
| **Lack of Premeditation** | **7.78** | **2.56** | **7.70** | **2.34** | **1.38** |
| **Lack of Perseverance** | **7.26** | **2.44** | **6.95** | **2.20** | **5.48^***^** |
| **Negative Urgency** | **8.66** | **2.85** | **8.41** | **2.61** | **3.75^***^** |
| **Sensation Seeking** | **9.50** | **2.84** | **9.83** | **2.67** | **-5.07^***^** |
| **Behavioral Inhibition** | **9.98** | **3.92** | **9.32** | **3.70** | **7.28^***^** |
| **Depression** | **58.09** | **6.96** | **52.04** | **3.77** | **40.00^***^** |
| **Somatic** | **58.84** | **7.55** | **54.36** | **5.79** | **26.40^***^** |
| **ADHD** | **56.51** | **7.21** | **52.10** | **4.25** | **27.97^***^** |
| **Sluggish Cognitive Tempo** | **55.94** | **6.98** | **52.02** | **4.21** | **25.62^***^** |
| **Oppositional defiance** | **56.65** | **6.93** | **52.42** | **4.17** | **27.85^***^** |
| **Conduct disorder** | **55.74** | **7.30** | **52.16** | **4.43** | **22.41^***^** |
| **Stress indicators** |  | |  | |  |
| **Demographics** |  | |  | |  |
| **Parents Education** | **3.49** | **1.15** | **3.56** | **1.18** | **-2.46^*^** |
| **Family income** | **1.87** | **0.98** | **1.97** | **1.00** | **-4.03^**^** |
| **Body mass index** | **18.92** | **4.36** | **18.76** | **4.13** | **1.58** |
| **Area Deprivation Index** | **41.39** | **27.08** | **39.54** | **26.96** | **2.81^**^** |
|  | N | % | N | % |  |
| **Recent social deprivation** | **646** | **28.5** | **1758** | **18.9** | **9.49^***^** |
| **Physical health** |  | |  | |  |
|  | Mean | S.D. | Mean | S.D. |  |
| **Initiating and maintaining sleep disorders** | **13.75** | **4.29** | **11.08** | **3.16** | **27.77^***^** |
| **Sleep breathing disorders** | **3.97** | **1.40** | **3.71** | **1.18** | **8.20^***^** |
| **Disorders of arousal or nightmares** | **3.75** | **1.17** | **3.34** | **0.76** | **15.95^***^** |
| **Sleep wake transition disorders** | **9.34** | **3.11** | **7.76** | **2.28** | **22.70^***^** |
| **Excessive somnolence disorders** | **8.00** | **2.90** | **6.59** | **2.09** | **21.84^***^** |
| **Sleep hyperhidrosis** | **2.76** | **1.56** | **2.33** | **0.99** | **12.31^***^** |
| **Screen time during weekdays** | **3.65** | **3.17** | **3.40** | **3.07** | **3.39^**^** |
| **Physical activity** | **3.34** | **2.29** | **3.53** | **2.32** | **-3.51^***^** |
|  | N | % | N | % |  |
| Substance use | 541 | 23.9 | 2136 | 23.0 | 0.89 |
| **Environment** |  | |  | |  |
| Family |  | |  | |  |
|  | Mean | S.D. | Mean | S.D. |  |
| Multi-group ethnic identity (caregiver) | 3.34 | 0.88 | 3.33 | 0.89 | 0.42 |
| **Neighborhood safety** | **3.79** | **0.99** | **3.92** | **0.96** | **-5.95^***^** |
| **Family conflict** | **2.19** | **2.01** | **2.00** | **1.93** | **4.11^***^** |
| **Prosocial behavior (child report)** | **1.66** | **0.38** | **1.68** | **0.37** | **-2.51^*^** |
| **Prosocial behavior (parent report)** | **1.68** | **0.43** | **1.77** | **0.39** | **-9.15^***^** |
| **Acceptance by parent** | **2.77** | **0.31** | **2.78** | **0.30** | **-2.50^*^** |
| School |  | |  | |  |
| **School environment** | **19.67** | **3.02** | **20.02** | **2.76** | **-5.05^***^** |
| **School disengagement** | **3.83** | **1.52** | **3.71** | **1.44** | **3.32^**^** |
| Friends(girls) | 3.33 | 6.89 | 3.35 | 6.34 | -0.79 |
| Friends(boys) | 2.93 | 5.72 | 3.03 | 5.60 | -0.17 |
| **Mental health** |  | |  | |  |
| **Traumatic history** | **0.69** | **1.00** | **0.44** | **0.86** | **10.52^***^** |

^***^, *p*<0.001

^**^, *p*<0.01

^*^, *p*<0.05

## Table S**7** Baseline factors differentiating subthreshold anxiety from healthy controls (CBCL).

|  | Odds ratio | 95%CIs | | *β* | *z* | *Wald statistic* | *p* |
| --- | --- | --- | --- | --- | --- | --- | --- |
|  |  | Lower | Upper |  |  |  |  |
| **Diathesis indicators** |  |  |  |  |  |  |  |
| **Demographics** |  |  |  |  |  |  |  |
| Age | 0.95 | 0.90 | 1.01 | -0.05 | -1.80 | 3.09 | 0.07 |
| Female | 1.13 | 1.00 | 1.29 | 0.12 | 1.89 | 3.57 | 0.06 |
| Grandparents’ psychiatric history | 1.06 | 0.88 | 1.27 | 0.05 | 0.50 | 0.34 | 0.62 |
| Parents’ psychiatric history | 1.23 | 1.04 | 1.46 | 0.21 | 2.40 | 5.58 | <0.05 |
| Grandparents and parents’ psychiatric history | 1.25 | 1.08 | 1.44 | 0.22 | 3.08 | 9.4 | <0.05 |
| Left-handedness | 1.38 | 1.13 | 1.69 | 0.33 | 3.18 | 10.09 | <0.05 |
| Mixed-handedness | 0.93 | 0.79 | 1.1 | -0.07 | -0.82 | 0.68 | 0.41 |
| **Mental health** |  |  |  |  |  |  |  |
| Prodromal Psychosis | 1.01 | 0.94 | 1.08 | 0.01 | 0.28 | 0.04 | 0.78 |
| Mean severity of Prodromal Psychosis | 1.01 | 0.94 | 1.09 | 0.01 | 0.20 | 0.16 | 0.84 |
| Lack of Premeditation | 0.9 | 0.84 | 0.96 | -0.11 | -3.13 | 9.73 | <0.05 |
| Lack of Perseverance | 0.98 | 0.92 | 1.04 | -0.02 | -0.65 | 0.39 | 0.51 |
| Negative Urgency | 0.95 | 0.90 | 1.01 | -0.05 | -1.47 | 2.21 | 0.14 |
| **Sensation Seeking** | **0.85** | **0.80** | **0.90** | **-0.16** | **-5.49** | **30.35^***^** | **<0.001** |
| **Behavioral Inhibition** | **1.22** | **1.15** | **1.3** | **0.2** | **6.70** | **44.72^***^** | **<0.001** |
| **Depression** | **1.87** | **1.75** | **1.99** | **0.62** | **19.44** | **377.71^***^** | **<0.001** |
| **Somatic** | **1.18** | **1.11** | **1.26** | **0.16** | **5.09** | **25.54^***^** | **<0.001** |
| **ADHD** | **1.38** | **1.31** | **1.45** | **0.32** | **11.70** | **137.07^***^** | **<0.001** |
| **Sluggish Cognitive Tempo** | **1.18** | **1.11** | **1.25** | **0.16** | **5.63** | **31.92^***^** | **<0.001** |
| **Oppositional defiance** | **1.29** | **1.20** | **1.39** | **0.26** | **6.92** | **48.3^***^** | **<0.001** |
| Conduct disorder | 0.99 | 0.92 | 1.06 | -0.01 | -0.23 | 0.07 | 0.82 |
| **Stress indicators** |  |  |  |  |  |  |  |
| **Demographics** |  |  |  |  |  |  |  |
| Parents Education | 1.05 | 0.98 | 1.13 | 0.05 | 1.02 | 1.74 | 0.31 |
| Family income | 0.98 | 0.91 | 1.06 | -0.02 | -0.22 | 0.18 | 0.82 |
| Recent social deprivation | 1.04 | 0.89 | 1.21 | 0.04 | 0.55 | 0.25 | 0.58 |
| Area Deprivation Index | 0.97 | 0.90 | 1.03 | -0.03 | -1.46 | 0.93 | 0.14 |
| Body mass index | 0.98 | 0.92 | 1.03 | -0.02 | -0.80 | 0.68 | 0.42 |
| **Physical health** |  |  |  |  |  |  |  |
| **Initiating and maintaining sleep disorders** | **1.21** | **1.14** | **1.28** | **0.19** | **6.28** | **38.87^***^** | **<0.001** |
| Sleep breathing disorders | 0.93 | 0.88 | 0.99 | -0.07 | -2.37 | 5.65 | <0.05 |
| **Disorders of arousal or nightmares** | **1.13** | **1.07** | **1.19** | **0.12** | **4.47** | **19.91^***^** | **<0.001** |
| **Sleep wake transition disorders** | **1.22** | **1.15** | **1.29** | **0.20** | **6.60** | **43.26^***^** | **<0.001** |
| Excessive somnolence disorders | 0.93 | 0.88 | 0.99 | -0.07 | -2.30 | 5.42 | <0.05 |
| Sleep hyperhidrosis | 1.07 | 1.01 | 1.12 | 0.06 | 2.44 | 5.97 | <0.05 |
| Screen time during weekdays | 0.96 | 0.90 | 1.02 | -0.04 | -1.22 | 1.78 | 0.22 |
| Physical activity | 0.99 | 0.94 | 1.05 | -0.01 | -0.37 | 0.07 | 0.71 |
| Substance use | 0.99 | 0.86 | 1.13 | -0.01 | -0.21 | 0.03 | 0.83 |
| **Environment** |  |  |  |  |  |  |  |
| Family |  |  |  |  |  |  |  |
| Multi-group ethnic identity (caregiver) | 1.03 | 0.97 | 1.09 | 0.03 | 1.34 | 0.86 | 0.18 |
| Neighborhood safety | 1.03 | 0.97 | 1.1 | 0.03 | 0.89 | 0.99 | 0.37 |
| **Family conflict** | **1.09** | **1.02** | **1.16** | **0.08** | **5.24** | **8.26^*^** | **<0.01** |
| Prosocial behavior (child report) | 1.07 | 1.01 | 1.14 | 0.07 | 2.32 | 5.37 | <0.05 |
| Prosocial behavior (parent report) | 0.92 | 0.86 | 0.98 | -0.09 | -2.61 | 6.91 | <0.05 |
| Acceptance by parent | 1.03 | 0.97 | 1.09 | 0.03 | 0.70 | 0.77 | 0.48 |
| School |  |  |  |  |  |  |  |
| School environment | 0.97 | 0.91 | 1.03 | -0.03 | -1.05 | 1.26 | 0.29 |
| School disengagement | 1.00 | 0.95 | 1.07 | 0 | 0.08 | 0.01 | 0.93 |
| Friends(girls) | 1.00 | 0.93 | 1.06 | 0 | -0.14 | 0.02 | 0.89 |
| Friends(boys) | 0.98 | 0.91 | 1.04 | -0.02 | -0.78 | 0.54 | 0.43 |
| **Mental health** |  |  |  |  |  |  |  |
| Traumatic history | 1.03 | 0.98 | 1.09 | 0.03 | 1.06 | 1.49 | 0.29 |

^***^, *p*<0.001 following Bonferroni correction.

^**^, *p*<0.01 following Bonferroni correction.

^*^, *p*<0.05 following Bonferroni correction.

## Table S**8** Risk and protective factors of developing an anxiety disorder (CBCL).

|  | Odds ratio | 95%CIs | | *β* | *z* | Wald statistic | *p* |
| --- | --- | --- | --- | --- | --- | --- | --- |
|  |  | Lower | Upper |  |  |  |  |
| **Diathesis indicators** |  |  |  |  |  |  |  |
| **Demographics** |  |  |  |  |  |  |  |
| Age | 1.03 | 0.93 | 1.21 | 0.03 | 0.46 | 0.13 | 0.71 |
| Female | 1.19 | 0.83 | 1.54 | 0.18 | 0.97 | 1.08 | 0.30 |
| Grandparents’ psychiatric history | 1.58 | 0.98 | 2.88 | 0.46 | 1.68 | 2.59 | 0.11 |
| **Parents’ psychiatric history** | **3.00** | **1.90** | **4.82** | **1.10** | **4.55** | **19.81^***^** | **<0.001** |
| **Grandparents and parents’ psychiatric history** | **5.42** | **3.92** | **8.66** | **1.69** | **7.82** | **64.60^***^** | **<0.001** |
| Left-handedness | 1.00 | 0.54 | 1.61 | 0.00 | -0.04 | 0.00 | 0.99 |
| Mixed-handedness | 0.93 | 0.59 | 1.28 | -0.07 | -0.46 | 0.13 | 0.72 |
| **Mental health** |  |  |  |  |  |  |  |
| Prodromal Psychosis | 1.16 | 1.00 | 1.34 | 0.15 | 1.73 | 3.56 | 0.06 |
| Mean severity of Prodromal Psychosis | 1.25 | 1.02 | 1.40 | 0.22 | 2.44 | 6.83 | <0.05 |
| **Lack of Premeditation** | **0.72** | **0.62** | **0.84** | **-0.32** | **-3.91** | **14.20^**^** | **<0.001** |
| **Lack of Perseverance** | **1.36** | **1.19** | **1.59** | **0.31** | **3.97** | **16.20^**^** | **<0.001** |
| Negative Urgency | 1.10 | 0.89 | 1.16 | 0.09 | 0.96 | 1.56 | 0.21 |
| **Conduct disorder** | **1.54** | **1.60** | **1.99** | **0.43** | **6.74** | **51.11^***^** | **<0.001** |
| **Stress indicators** |  |  |  |  |  |  |  |
| **Demographics** |  |  |  |  |  |  |  |
| Parents Education | 0.96 | 0.84 | 1.16 | -0.04 | -0.15 | 0.20 | 0.66 |
| Family income | 0.88 | 0.73 | 1.04 | -0.13 | -1.34 | 2.05 | 0.15 |
| Recent social deprivation | 1.24 | 0.93 | 1.87 | 0.21 | 1.09 | 1.29 | 0.26 |
| **Area Deprivation Index** | **1.32** | **1.19** | **1.46** | **0.28** | **3.64** | **13.27^**^** | **<0.001** |
| Body mass index | 1.08 | 0.93 | 1.24 | 0.08 | 1.03 | 1.12 | 0.29 |
| **Physical health** |  |  |  |  |  |  |  |
| Sleep breathing disorders | 1.03 | 0.90 | 1.17 | 0.03 | 0.36 | 0.24 | 0.63 |
| **Excessive somnolence disorders** | **1.73** | **1.54** | **1.94** | **0.55** | **8.88** | **83.87^***^** | **<0.001** |
| **Sleep hyperhidrosis** | **1.32** | **1.19** | **1.46** | **0.28** | **5.61** | **29.4^***^** | **<0.001** |
| Screen time during weekdays | 0.97 | 0.83 | 1.13 | -0.03 | -0.47 | 0.13 | 0.72 |
| Physical activity | 0.96 | 0.82 | 1.09 | -0.05 | -0.73 | 0.40 | 0.53 |
| Substance use | 0.94 | 0.66 | 1.28 | -0.06 | -0.58 | 0.14 | 0.70 |
| **Environment** |  |  |  |  |  |  |  |
| Family |  |  |  |  |  |  |  |
| Multi-group ethnic identity (caregiver) | 0.94 | 0.82 | 1.09 | -0.06 | -1.15 | 0.76 | 0.38 |
| Neighborhood safety | 1.10 | 0.95 | 1.28 | 0.09 | 1.41 | 1.38 | 0.24 |
| Prosocial behavior (child report) | 0.83 | 0.91 | 1.21 | -0.18 | 0.50 | 5.77 | <0.05 |
| Prosocial behavior (parent report) | 1.05 | 0.93 | 1.30 | 0.04 | 1.14 | 0.36 | 0.55 |
| Acceptance by parent | 1.10 | 0.94 | 1.26 | 0.10 | 1.13 | 1.27 | 0.26 |
| School |  |  |  |  |  |  |  |
| **School environment** | **0.84** | **0.73** | **0.97** | **-0.18** | **-2.61** | **6.18^*^** | **<0.01** |
| School disengagement | 1.02 | 0.88 | 1.18 | 0.02 | 0.13 | 0.07 | 0.80 |
| Friends(girls) | 1.04 | 0.90 | 1.19 | 0.04 | 0.15 | 0.29 | 0.59 |
| Friends(boys) | 0.90 | 0.72 | 1.08 | -0.10 | -0.74 | 0.98 | 0.32 |
| **Mental health** |  |  |  |  |  |  |  |
| Traumatic history | 1.08 | 0.97 | 1.19 | 0.08 | 1.40 | 2.26 | 0.13 |

^***^, *p*<0.001 following Bonferroni correction.

^**^, *p*<0.01 following Bonferroni correction.

^*^, *p*<0.05 following Bonferroni correction.

## Table S**9** Risk and protective factors of remained subthreshold anxiety (CBCL).

|  | Odds ratio | 95%CIs | | *β* | *z* | Wald statistic | *p* |
| --- | --- | --- | --- | --- | --- | --- | --- |
|  |  | Lower | Upper |  |  |  |  |
| **Diathesis indicators** |  |  |  |  |  |  |  |
| **Demographics** |  |  |  |  |  |  |  |
| Age | 1.1 | 0.95 | 1.28 | 0.1 | 1.50 | 1.73 | 0.19 |
| **Female** | **1.42** | **1.02** | **1.97** | **0.35** | **2.06** | **4.43^*^** | **<0.01** |
| Grandparents’ psychiatric history | 1.33 | 0.81 | 2.15 | 0.28 | 1.14 | 1.29 | 0.26 |
| Parents’ psychiatric history | 1.74 | 1.09 | 2.76 | 0.56 | 2.46 | 5.57 | <0.05 |
| **Grandparents and parents’ psychiatric history** | **2.35** | **1.63** | **3.44** | **0.85** | **4.75** | **20.11^***^** | **<0.001** |
| Left-handedness | 0.81 | 0.41 | 1.47 | -0.21 | -0.75 | 0.44 | 0.51 |
| Mixed-handedness | 1.22 | 0.82 | 1.77 | 0.2 | 1.07 | 1.05 | 0.31 |
| **Mental health** |  |  |  |  |  |  |  |
| Prodromal Psychosis | 1.09 | 0.92 | 1.28 | 0.08 | 0.72 | 0.96 | 0.33 |
| Mean severity of Prodromal Psychosis | 1.13 | 0.94 | 1.34 | 0.12 | 1.45 | 1.76 | 0.18 |
| Lack of Premeditation | 0.85 | 0.71 | 1 | -0.17 | -1.85 | 3.56 | 0.06 |
| Lack of Perseverance | 1 | 0.84 | 1.18 | 0 | -0.22 | 0 | 0.96 |
| Negative Urgency | 1.16 | 1 | 1.35 | 0.15 | 1.49 | 3.84 | 0.05 |
| **Conduct disorder** | **1.31** | **1.15** | **1.5** | **0.27** | **3.86** | **16.31^**^** | **<0.001** |
| **Stress indicators** |  |  |  |  |  |  |  |
| **Demographics** |  |  |  |  |  |  |  |
| Parents Education | 1.08 | 0.91 | 1.3 | 0.08 | 1.01 | 0.81 | 0.37 |
| Family income | 0.97 | 0.79 | 1.18 | -0.04 | -0.40 | 0.13 | 0.72 |
| Recent social deprivation | 1.14 | 0.77 | 1.69 | 0.13 | 0.55 | 0.45 | 0.50 |
| Area Deprivation Index | 0.83 | 0.7 | 0.99 | -0.18 | -1.75 | 4.33 | 0.04 |
| Body mass index | 1.13 | 0.98 | 1.3 | 0.12 | 1.28 | 3.02 | 0.08 |
| **Physical health** |  |  |  |  |  |  |  |
| Sleep breathing disorders | 1.06 | 0.92 | 1.21 | 0.06 | 0.78 | 0.7 | 0.40 |
| **Excessive somnolence disorders** | **1.46** | **1.28** | **1.67** | **0.38** | **5.48** | **32.92^***^** | **<0.001** |
| **Sleep hyperhidrosis** | **1.22** | **1.08** | **1.37** | **0.2** | **3.29** | **10.96^*^** | **<0.001** |
| Screen time during weekdays | 0.98 | 0.84 | 1.13 | -0.03 | -0.54 | 0.11 | 0.74 |
| **Physical activity** | **0.83** | **0.71** | **0.96** | **-0.19** | **-3.15** | **8.23^*^** | **<0.01** |
| Substance use | 0.82 | 0.57 | 1.16 | -0.2 | -1.17 | 1.24 | 0.27 |
| **Environment** |  |  |  |  |  |  |  |
| Family |  |  |  |  |  |  |  |
| Multi-group ethnic identity (caregiver) | 1.05 | 0.91 | 1.22 | 0.05 | 1.24 | 0.5 | 0.48 |
| Neighborhood safety | 0.9 | 0.77 | 1.04 | -0.11 | -1.35 | 2.15 | 0.14 |
| Prosocial behavior (child report) | 0.87 | 0.76 | 1 | -0.14 | -2.10 | 4.13 | <0.05 |
| Prosocial behavior (parent report) | 0.93 | 0.79 | 1.09 | -0.08 | -0.84 | 0.84 | 0.36 |
| Acceptance by parent | 1.12 | 0.96 | 1.32 | 0.12 | 2.07 | 1.99 | 0.16 |
| School |  |  |  |  |  |  |  |
| **School environment** | **0.79** | **0.68** | **0.93** | **-0.23** | **-2.85** | **8.76^*^** | **<0.01** |
| School disengagement | 1.03 | 0.89 | 1.2 | 0.03 | 0.28 | 0.19 | 0.66 |
| Friends(girls) | 0.99 | 0.87 | 1.12 | -0.01 | -0.03 | 0.01 | 0.92 |
| Friends(boys) | 1.15 | 1.03 | 1.28 | 0.14 | 2.61 | 6.84 | <0.01 |
| **Mental health** |  |  |  |  |  |  |  |
| Traumatic history | 1.07 | 0.95 | 1.19 | 0.07 | 0.86 | 1.64 | 0.20 |

^***^, *p*<0.001 following Bonferroni correction.

^**^, *p*<0.01 following Bonferroni correction.

^*^, *p*<0.05 following Bonferroni correction.

## Table S**10** Risk and protective factors of remission from subthreshold anxiety (CBCL).

|  | Odds ratio | 95%CIs | | *β* | *z* | Wald statistic | *p* |
| --- | --- | --- | --- | --- | --- | --- | --- |
|  |  | Lower | Upper |  |  |  |  |
| **Diathesis indicators** |  |  |  |  |  |  |  |
| **Demographics** |  |  |  |  |  |  |  |
| Age | 1.31 | 1.09 | 1.58 | 0.27 | 2.80 | 7.88 | <0.01 |
| Female | 0.91 | 0.57 | 1.46 | -0.09 | -0.49 | 0.15 | 0.70 |
| Grandparents’ psychiatric history | 1.13 | 0.55 | 2.34 | 0.12 | 0.19 | 0.11 | 0.74 |
| Parents' psychiatric history | 1.98 | 1.04 | 3.79 | 0.68 | 2.15 | 4.29 | 0.04 |
| **Grandparents and parents’ psychiatric history** | **3.13** | **1.82** | **5.45** | **1.14** | **4.04** | **16.69^**^** | **<0.001** |
| Left-handedness | 1.03 | 0.47 | 2.29 | 0.03 | 0.02 | 0 | 0.95 |
| Mixed-handedness | 1.11 | 0.64 | 1.93 | 0.1 | 0.25 | 0.13 | 0.71 |
| **Mental health** |  |  |  |  |  |  |  |
| Prodromal Psychosis | 1.4 | 1.11 | 1.79 | 0.33 | 2.62 | 7.57 | <0.01 |
| Mean severity of Prodromal Psychosis | 0.94 | 0.75 | 1.17 | -0.06 | -0.31 | 0.34 | 0.56 |
| Lack of Premeditation | 0.84 | 0.67 | 1.06 | -0.17 | -1.53 | 2.22 | 0.14 |
| Lack of Perseverance | 1.31 | 1.06 | 1.61 | 0.27 | 2.37 | 6.42 | 0.01 |
| Negative Urgency | 1.02 | 0.83 | 1.24 | 0.02 | 0.08 | 0.02 | 0.88 |
| Conduct disorder | 0.99 | 0.85 | 1.16 | -0.01 | -0.31 | 0.01 | 0.92 |
| **Stress indicators** |  |  |  |  |  |  |  |
| **Demographics** |  |  |  |  |  |  |  |
| Parents Education | 1.29 | 1.02 | 1.63 | 0.25 | 2.04 | 4.36 | <0.05 |
| Family income | 0.74 | 0.57 | 0.96 | -0.3 | -2.02 | 5 | <0.05 |
| Recent social deprivation | 0.88 | 0.52 | 1.49 | -0.12 | -0.32 | 0.22 | 0.64 |
| Area Deprivation Index | 0.71 | 0.57 | 0.89 | -0.34 | -2.90 | 8.72 | <0.01 |
| Body mass index | 0.99 | 0.82 | 1.21 | -0.01 | 0.35 | 0.01 | 0.94 |
| **Physical health** |  |  |  |  |  |  |  |
| Sleep breathing disorders | 1.1 | 0.91 | 1.32 | 0.09 | 1.03 | 0.97 | 0.33 |
| Excessive somnolence disorders | 1.28 | 1.08 | 1.51 | 0.24 | 2.91 | 8.35 | <0.01 |
| Sleep hyperhidrosis | 1.21 | 1.05 | 1.41 | 0.19 | 2.51 | 6.24 | <0.05 |
| Screen time during weekdays | 1.03 | 0.84 | 1.27 | 0.03 | 0.17 | 0.11 | 0.74 |
| Physical activity | 0.85 | 0.7 | 1.04 | -0.16 | -1.66 | 2.51 | 0.11 |
| Substance use | 0.77 | 0.49 | 1.23 | -0.26 | -0.93 | 1.18 | 0.28 |
| **Environment** |  |  |  |  |  |  |  |
| Family |  |  |  |  |  |  |  |
| Multi-group ethnic identity (caregiver) | 0.99 | 0.82 | 1.19 | -0.01 | 0.01 | 0.02 | 0.89 |
| Neighborhood safety | 0.96 | 0.79 | 1.18 | -0.04 | -0.42 | 0.12 | 0.73 |
| Prosocial behavior (child report) | 0.95 | 0.76 | 1.17 | -0.06 | -0.54 | 0.27 | 0.60 |
| Prosocial behavior (parent report) | 1.08 | 0.87 | 1.34 | 0.07 | 0.52 | 0.46 | 0.50 |
| Acceptance by parent | 1.12 | 0.91 | 1.4 | 0.12 | 1.37 | 1.15 | 0.28 |
| School |  |  |  |  |  |  |  |
| School environment | 0.9 | 0.74 | 1.1 | -0.1 | -1.00 | 1.05 | 0.30 |
| School disengagement | 0.93 | 0.76 | 1.14 | -0.07 | -0.81 | 0.46 | 0.50 |
| Friends(girls) | 1.17 | 0.95 | 1.5 | 0.15 | 1.33 | 1.85 | 0.17 |
| Friends(boys) | 1.03 | 0.77 | 1.37 | 0.03 | 0.14 | 0.04 | 0.84 |
| **Mental health** |  |  |  |  |  |  |  |
| **Traumatic history** | **1.25** | **1.02** | **1.55** | **0.22** | **3.74** | **8.37^*^** | **<0.05** |

^***^, *p*<0.001 following Bonferroni correction.

^**^, *p*<0.01 following Bonferroni correction.

^*^, *p*<0.05 following Bonferroni correction.

## Table S**11** Baseline factors differentiating subthreshold anxiety from healthy controls (KSADS-5).

|  | Odds ratio | 95%CIs | | *β* | *z* | *Wald statistic* | *p* |
| --- | --- | --- | --- | --- | --- | --- | --- |
|  |  | Lower | Upper |  |  |  |  |
| **Diathesis indicators** |  |  |  |  |  |  |  |
| **Demographics** |  |  |  |  |  |  |  |
| Age | 1.03 | 0.98 | 1.07 | 0.03 | 1.16 | 1.35 | 0.25 |
| Female | 1.16 | 1.04 | 1.28 | 0.15 | 2.79 | 7.8 | <0.01 |
| Grandparents’ psychiatric history | 1.22 | 1.07 | 1.4 | 0.2 | 2.92 | 8.54 | <0.01 |
| Parents’ psychiatric history | 1.08 | 0.94 | 1.24 | 0.08 | 1.14 | 1.3 | 0.25 |
| Grandparents and parents’ psychiatric history | 1.19 | 1.07 | 1.34 | 0.18 | 3.06 | 9.35 | <0.01 |
| Left-handedness | 0.93 | 0.78 | 1.1 | -0.08 | -0.87 | 0.76 | 0.38 |
| Mixed-handedness | 1.09 | 0.96 | 1.24 | 0.09 | 1.38 | 1.89 | 0.17 |
| **Mental health** |  |  |  |  |  |  |  |
| Prodromal Psychosis | 1.02 | 0.96 | 1.08 | 0.02 | 0.72 | 0.51 | 0.47 |
| Mean severity of Prodromal Psychosis | 0.96 | 0.91 | 1.02 | -0.04 | -1.26 | 1.58 | 0.21 |
| Lack of Premeditation | 1 | 0.95 | 1.05 | 0 | -0.07 | 0 | 0.95 |
| Lack of Perseverance | 1.01 | 0.96 | 1.07 | 0.01 | 0.5 | 0.25 | 0.62 |
| Negative Urgency | 0.99 | 0.95 | 1.04 | -0.01 | -0.26 | 0.07 | 0.79 |
| **Sensation Seeking** | **0.85** | **0.81** | **0.89** | **-0.16** | **-6.79** | **46.13^***^** | **<0.001** |
| **Behavioral Inhibition** | **1.15** | **1.09** | **1.2** | **0.14** | **5.67** | **32.2^***^** | **<0.001** |
| Depression | 1.07 | 1.01 | 1.14 | 0.07 | 2.44 | 5.95 | <0.05 |
| **Somatic** | **1.16** | **1.11** | **1.22** | **0.15** | **6.43** | **41.35^***^** | **<0.001** |
| **ADHD** | **1.11** | **1.05** | **1.18** | **0.11** | **3.64** | **13.24^*^** | **<0.001** |
| Sluggish Cognitive Tempo | 1.06 | 1.01 | 1.12 | 0.06 | 2.33 | 5.45 | <0.05 |
| **Oppositional defiance** | **1.13** | **1.06** | **1.21** | **0.12** | **3.67** | **13.48^*^** | **<0.001** |
| Conduct disorder | 0.93 | 0.87 | 0.99 | -0.07 | -2.31 | 5.36 | <0.05 |
| **Stress indicators** |  |  |  |  |  |  |  |
| **Demographics** |  |  |  |  |  |  |  |
| Parents Education | 1.04 | 0.98 | 1.1 | 0.04 | 1.38 | 1.91 | 0.17 |
| Family income | 0.99 | 0.93 | 1.05 | -0.01 | -0.43 | 0.18 | 0.67 |
| **Recent social deprivation** | **1.26** | **1.12** | **1.43** | **0.23** | **3.72** | **13.81^*^** | **<0.001** |
| Area Deprivation Index | 0.97 | 0.91 | 1.04 | -0.03 | -0.81 | 0.66 | 0.42 |
| Body mass index | 0.98 | 0.93 | 1.02 | -0.02 | -0.95 | 0.91 | 0.34 |
| **Physical health** |  |  |  |  |  |  |  |
| **Initiating and maintaining sleep disorders** | **1.15** | **1.09** | **1.21** | **0.14** | **5.17** | **26.75^***^** | **<0.001** |
| Sleep breathing disorders | 0.99 | 0.95 | 1.04 | -0.01 | -0.4 | 0.16 | 0.69 |
| Disorders of arousal or nightmares | 1.06 | 1.01 | 1.11 | 0.06 | 2.49 | 6.22 | <0.05 |
| **Sleep wake transition disorders** | **1.09** | **1.04** | **1.15** | **0.09** | **3.39** | **11.48^*^** | **<0.001** |
| Excessive somnolence disorders | 1.07 | 1.02 | 1.12 | 0.07 | 2.54 | 6.46 | <0.05 |
| **Sleep hyperhidrosis** | **1.1** | **1.05** | **1.15** | **0.09** | **4.12** | **16.97^**^** | **<0.001** |
| **Screen time during weekdays** | **1.09** | **1.04** | **1.14** | **0.09** | **3.48** | **12.1^*^** | **<0.001** |
| Physical activity | 1.03 | 0.98 | 1.08 | 0.03 | 1.14 | 1.29 | 0.26 |
| Substance use | 1.01 | 0.9 | 1.12 | 0.01 | 0.14 | 0.02 | 0.89 |
| **Environment** |  |  |  |  |  |  |  |
| Family |  |  |  |  |  |  |  |
| Multi-group ethnic identity (caregiver) | 1.01 | 0.96 | 1.05 | 0.01 | 0.28 | 0.08 | 0.78 |
| Neighborhood safety | 0.97 | 0.92 | 1.02 | -0.03 | -1.19 | 1.41 | 0.24 |
| Family conflict | 0.95 | 0.9 | 0.99 | -0.05 | -2.19 | 4.78 | <0.05 |
| Prosocial behavior (child report) | 0.97 | 0.93 | 1.02 | -0.03 | -1.08 | 1.17 | 0.28 |
| Prosocial behavior (parent report) | 1.04 | 0.98 | 1.09 | 0.04 | 1.36 | 1.84 | 0.18 |
| Acceptance by parent | 1.03 | 0.98 | 1.09 | 0.03 | 1.27 | 1.62 | 0.20 |
| School |  |  |  |  |  |  |  |
| School environment | 0.95 | 0.91 | 1 | -0.05 | -1.85 | 3.41 | 0.06 |
| School disengagement | 0.98 | 0.94 | 1.03 | -0.02 | -0.71 | 0.5 | 0.48 |
| Friends(girls) | 1.01 | 0.96 | 1.06 | 0.01 | 0.3 | 0.09 | 0.77 |
| Friends(boys) | 1 | 0.95 | 1.04 | 0 | -0.17 | 0.03 | 0.87 |
| **Mental health** |  |  |  |  |  |  |  |
| **Traumatic history** | **1.08** | **1.03** | **1.13** | **0.08** | **3.45** | **11.88^*^** | **<0.001** |

^***^, *p*<0.001 following Bonferroni correction.

^**^, *p*<0.01 following Bonferroni correction.

^*^, *p*<0.05 following Bonferroni correction.

## Table S**12** Significant risk and protective factors of progression into anxiety disorder (KSADS-5).

|  | Odds ratio | 95%CIs | | *β* | *z* | Wald statistic | *p* |
| --- | --- | --- | --- | --- | --- | --- | --- |
|  |  | Lower | Upper |  |  |  |  |
| Female | 1.61 | 1.07 | 2.42 | 0.48 | 2.30 | 5.27 | <0.05 |
| Parents’ psychiatric history | 2.33 | 1.33 | 4.12 | 0.85 | 2.96 | 8.75 | <0.01 |
| **Grandparents and parents’ psychiatric history** | **2.67** | **1.65** | **4.44** | **0.98** | **3.90** | **15.23^**^** | **<0.001** |
| Prodromal Psychosis | 1.31 | 1.06 | 1.59 | 0.27 | 2.61 | 6.79 | <0.01 |
| **Depression** | **1.51** | **1.26** | **1.82** | **0.42** | **4.46** | **19.86^***^** | **<0.001** |
| **Disorders of arousal or nightmares** | **1.30** | **1.15** | **1.48** | **0.26** | **4.06** | **16.52^**^** | **<0.001** |

^***^, *p*<0.001 following Bonferroni correction.

^**^, *p*<0.01 following Bonferroni correction.

^*^, *p*<0.05 following Bonferroni correction.

## Table S**13** Significant risk and protective factors of remained subthreshold anxiety (KSADS-5).

|  | Odds ratio | 95%CIs | | β | z | Wald statistic | p |
| --- | --- | --- | --- | --- | --- | --- | --- |
|  |  | Lower | Upper |  |  |  |  |
| Female | 1.33 | 1.10 | 1.62 | 0.29 | 2.90 | 8.43 | <0.01 |
| Grandparents’ psychiatric history | 1.35 | 1.05 | 1.74 | 0.30 | 2.32 | 5.37 | <0.05 |
| **Grandparents and parents’ psychiatric history** | **1.52** | **1.23** | **1.88** | **0.42** | **3.92** | **15.34^**^** | **<0.001** |
| Prodromal Psychosis | 1.12 | 1.00 | 1.25 | 0.11 | 2.06 | 4.24 | <0.05 |
| **Depression** | **1.27** | **1.15** | **1.40** | **0.24** | **4.74** | **22.46^***^** | **<0.001** |
| **Sluggish Cognitive Tempo** | **1.16** | **1.07** | **1.27** | **0.15** | **3.43** | **11.75^*^** | **<0.001** |
| Parents Education | 1.12 | 1.01 | 1.25 | 0.11 | 2.08 | 4.33 | <0.05 |
| Area Deprivation Index | 0.85 | 0.75 | 0.96 | -0.17 | -2.63 | 6.92 | <0.01 |
| Disorders of arousal or nightmares | 1.14 | 1.05 | 1.23 | 0.13 | 3.17 | 10.06 | <0.01 |
| **Excessive somnolence disorders** | **1.21** | **1.11** | **1.33** | **0.19** | **4.21** | **17.70^**^** | **<0.001** |

^***^, *p*<0.001 following Bonferroni correction.

^**^, *p*<0.01 following Bonferroni correction.

^*^, *p*<0.05 following Bonferroni correction.

## Table S**14** Significant risk and protective factors of remission from subthreshold anxiety (KSADS-5).

|  | Odds ratio | 95%CIs | | β | z | Wald statistic | p |
| --- | --- | --- | --- | --- | --- | --- | --- |
|  |  | Lower | Upper |  |  |  |  |
| **Grandparents and parents’ psychiatric history** | **2.45** | **1.44** | **4.30** | **0.90** | **3.23** | **12.42^**^** | **<0.001** |
| Lack of Perseverance | 1.26 | 1.03 | 1.54 | 0.23 | 2.23 | 4.96 | <0.05 |
| Depression | 1.23 | 1.01 | 1.48 | 0.20 | 2.10 | 4.40 | <0.05 |
| **School disengagement** | **1.31** | **1.07** | **1.60** | **0.27** | **2.59** | **8.71^*^** | **<0.01** |
| Friends(girls) | 1.16 | 1.00 | 1.35 | 0.15 | 2.09 | 4.37 | <0.05 |

^***^, *p*<0.001 following Bonferroni correction.

^**^, *p*<0.01 following Bonferroni correction.

^*^, *p*<0.05 following Bonferroni correction.

**
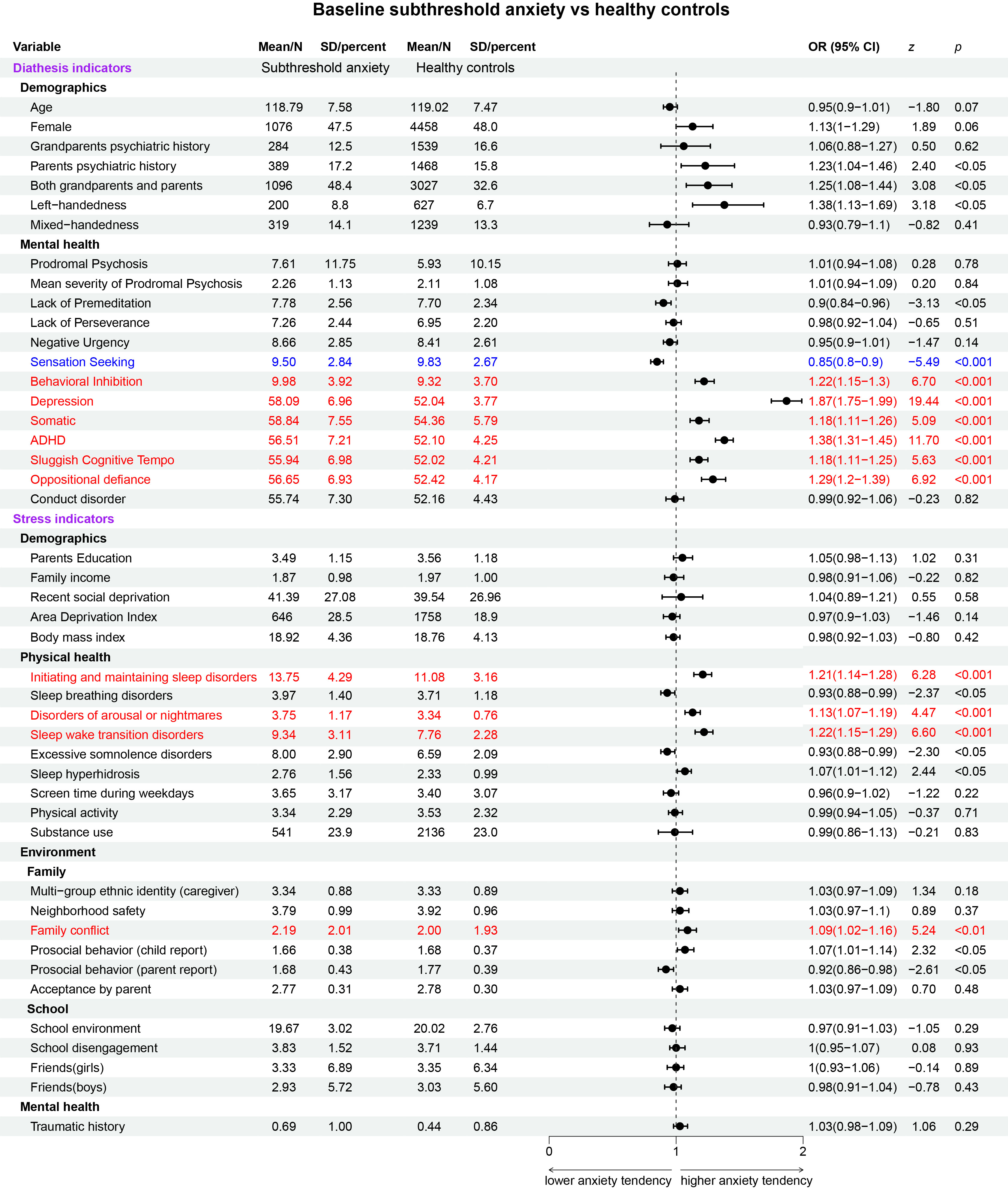
**

## Figure S**1** Association of higher anxiety symptoms with significant risk and protective factors illustrated through forest plot (Baseline subthreshold anxiety vs healthy controls).

The forest plot represents risk and protective factors of subthreshold anxiety, including behavioral inhibition, depression, somatic problem, ADHD, sluggish cognitive tempo, oppositional defiance, initiating and maintaining sleep disorders, disorders of arousal or nightmares, sleep wake transition disorders, family conflict, and sensation seeking. Red refers risk factors, which have higher anxiety tendency; blue refers protective factors, which have lower anxiety tendency.

## Data availability for production

Data used in the preparation of this article were obtained from the Adolescent Brain Cognitive Development^SM^ (ABCD) Study (<https://abcdstudy.org/>), held in the NIMH Data Archive (NDA).

## COI statement for production

All authors confirm that there are no conflicts of interest associated with this manuscript.

## Funding statement for production

This study was supported by National Key R & D Program of China (SIT2030-Major Projects 2022ZD0214300), Nature Science Foundation of China (ref: 32271139, 31900806), Guangdong Basic and Applied Basic Research Foundation (ref: 2023A1515011331), Science and Technology Program of Guangzhou, China (ref: 2023A04J1964), Guangzhou Philosophy and Social Science Project for 2022 Yangcheng Young Scholar during the fourteenth Five-year Plan Period (ref: 2022GZQN30). The funding organization played no further role in study design, data collection, analysis and interpretation, and paper writing.

## Supplementary References

Achenbach, T. M. (2011). Child Behavior Checklist. In J. S. Kreutzer, J. DeLuca, & B. Caplan (Eds.), *Encyclopedia of Clinical Neuropsychology* (pp. 546-552). New York, NY: Springer New York.

Arthur, M. W., Briney, J. S., Hawkins, J. D., Abbott, R. D., Brooke-Weiss, B. L., & Catalano, R. F. (2007). Measuring risk and protection in communities using the Communities That Care Youth Survey. *Eval Program Plann, 30*(2), 197-211. doi:10.1016/j.evalprogplan.2007.01.009

Asselmann, E., Wittchen, H. U., Lieb, R., Höfler, M., & Beesdo-Baum, K. (2014). Associations of fearful spells and panic attacks with incident anxiety, depressive, and substance use disorders: a 10-year prospective-longitudinal community study of adolescents and young adults. *J Psychiatr Res, 55*, 8-14. doi:10.1016/j.jpsychires.2014.04.001

Barch, D. M., Albaugh, M. D., Avenevoli, S., Chang, L., Clark, D. B., Glantz, M. D., . . . Sher, K. J. (2018). Demographic, physical and mental health assessments in the adolescent brain and cognitive development study: Rationale and description. *Developmental Cognitive Neuroscience, 32*, 55-66. doi:10.1016/j.dcn.2017.10.010

Bell, L. M., Verdezoto, C., Lardier, D. T., Herrera, A., Garcia-Reid, P., & Reid, R. J. (2025). Exploring the Role of Ethnic Identity, Attachment, and Family Prosocial Opportunities on BIPOC Adolescents' Anxiety and Depression. *J Racial Ethn Health Disparities*. doi:10.1007/s40615-025-02313-z

Blöte, A. W., Miers, A. C., Heyne, D. A., & Westenberg, P. M. (2015). Social Anxiety and the School Environment of Adolescents. In K. Ranta, A. M. La Greca, L.-J. Garcia-Lopez, & M. Marttunen (Eds.), *Social Anxiety and Phobia in Adolescents: Development, Manifestation and Intervention Strategies* (pp. 151-181). Cham: Springer International Publishing.

Bosman, R. C., Ten Have, M., de Graaf, R., Muntingh, A. D., van Balkom, A. J., & Batelaan, N. M. (2019). Prevalence and course of subthreshold anxiety disorder in the general population: A three-year follow-up study. *J Affect Disord, 247*, 105-113. doi:10.1016/j.jad.2019.01.018

Brener, N. D., Collins, J. L., Kann, L., Warren, C. W., & Williams, B. I. (1995). Reliability of the Youth Risk Behavior Survey Questionnaire. *American Journal of Epidemiology, 141*(6), 575-580. doi:10.1093/oxfordjournals.aje.a117473

Brown, S. A., Brumback, T., Tomlinson, K., Cummins, K., Thompson, W. K., Nagel, B. J., . . . Tapert, S. F. (2015). The National Consortium on Alcohol and NeuroDevelopment in Adolescence (NCANDA): A Multisite Study of Adolescent Development and Substance Use. *Journal of Studies on Alcohol and Drugs, 76*(6), 895-908. doi:10.15288/jsad.2015.76.895

Bruni, O., Ottaviano, S., Guidetti, V., Romoli, M., Innocenzi, M., Cortesi, F., & Giannotti, F. (1996). The Sleep Disturbance Scale for Children (SDSC) Construct ion and validation of an instrument to evaluate sleep disturbances in childhood and adolescence. *Journal of Sleep Research, 5*(4), 251-261. doi:<https://doi.org/10.1111/j.1365-2869.1996.00251.x>

Bruni, O., Ottaviano, S., Guidetti, V., Romoli, M., Innocenzi, M., Cortesi, F., & Giannotti, F. (1996). The Sleep Disturbance Scale for Children (SDSC). Construction and validation of an instrument to evaluate sleep disturbances in childhood and adolescence. *J Sleep Res, 5*(4), 251-261. doi:10.1111/j.1365-2869.1996.00251.x

Butler, D. C., Petterson, S., Phillips, R. L., & Bazemore, A. W. (2013). Measures of social deprivation that predict health care access and need within a rational area of primary care service delivery. *Health Serv Res, 48*(2 Pt 1), 539-559. doi:10.1111/j.1475-6773.2012.01449.x

Chellappa, S. L., & Aeschbach, D. (2022). Sleep and anxiety: From mechanisms to interventions. *Sleep Med Rev, 61*, 101583. doi:10.1016/j.smrv.2021.101583

Chen, K., Li, Q., Zhu, Z., Zhang, J., Niu, L., Dai, H., . . . Zhang, R. (2025). Risk and Protective Factors for the Evolution of Subthreshold Depression During Early Adolescence. *Journal of Adolescent Health, 76*(3), 385-395. doi:<https://doi.org/10.1016/j.jadohealth.2024.10.024>

Curtin, L. R., Mohadjer, L. K., Dohrmann, S. M., Kruszon-Moran, D., Mirel, L. B., Carroll, M. D., . . . Johnson, C. L. (2013). National Health and Nutrition Examination Survey: sample design, 2007-2010. *Vital Health Stat 2*(160), 1-23.

Dennis, E., Manza, P., & Volkow, N. D. (2022). Socioeconomic status, BMI, and brain development in children. *Transl Psychiatry, 12*(1), 33. doi:10.1038/s41398-022-01779-3

Echeverria, S. E., Diez-Roux, A. V., & Link, B. G. (2004). Reliability of self-reported neighborhood characteristics. *J Urban Health, 81*(4), 682-701. doi:10.1093/jurban/jth151

Goodman, R., Meltzer, H., & Bailey, V. (1998). The strengths and difficulties questionnaire: A pilot study on the validity of the self-report version. *European Child & Adolescent Psychiatry, 7*(3), 125-130. doi:10.1007/s007870050057

Goodman, R., Meltzer, H., & Bailey, V. (1998). The Strengths and Difficulties Questionnaire: a pilot study on the validity of the self-report version. . *European child & adolescent psychiatry*, 7(3), 125–130.

Greco, L. A., & Morris, T. L. (2005). Factors influencing the link between social anxiety and peer acceptance: Contributions of social skills and close friendships during middle childhood. *Behavior therapy, 36*(2), 197-205. doi:<https://doi.org/10.1016/S0005-7894(05)80068-1>

Herrington, H. M., Smith, T. B., Feinauer, E., & Griner, D. (2016). Reliability generalization of the Multigroup Ethnic Identity Measure-Revised (MEIM-R). *Journal of Counseling Psychology, 63*(5), 586-593. doi:10.1037/cou0000148

Jakuszkowiak-Wojten, K., Landowski, J., Wiglusz, M. S., & Cubała, W. J. (2015). Impulsivity in anxiety disorders. A critical review. *Psychiatr Danub, 27 Suppl 1*, S452-455.

Jorm, A. F., Christensen, H., Henderson, A. S., Jacomb, P. A., Korten, A. E., & Rodgers, B. (1998). Using the BIS/BAS scales to measure behavioural inhibition and behavioural activation: Factor structure, validity and norms in a large community sample. *Personality and Individual Differences, 26*(1), 49-58. doi:<https://doi.org/10.1016/S0191-8869(98)00143-3>

K, S. G. (2003). Area deprivation and widening inequalities in US mortality, 1969-1998. *American journal of public health*, 93(97), 1137–1143.

Kaufman, J., Birmaher, B., Brent, D., Rao, U., Flynn, C., Moreci, P., . . . Ryan, N. (1997). Schedule for Affective Disorders and Schizophrenia for School-Age Children-Present and Lifetime Version (K-SADS-PL): initial reliability and validity data. *J Am Acad Child Adolesc Psychiatry, 36*(7), 980-988. doi:10.1097/00004583-199707000-00021

Khan, A., Lee, E. Y., & Horwood, S. (2022). Adolescent screen time: associations with school stress and school satisfaction across 38 countries. *Eur J Pediatr, 181*(6), 2273-2281. doi:10.1007/s00431-022-04420-z

Khouja, J. N., Munafò, M. R., Tilling, K., Wiles, N. J., Joinson, C., Etchells, P. J., . . . Cornish, R. P. (2019). Is screen time associated with anxiety or depression in young people? Results from a UK birth cohort. *BMC Public Health, 19*(1), 82. doi:10.1186/s12889-018-6321-9

Koyuncu, A., Ayan, T., Ince Guliyev, E., Erbilgin, S., & Deveci, E. (2022). ADHD and Anxiety Disorder Comorbidity in Children and Adults: Diagnostic and Therapeutic Challenges. *Current Psychiatry Reports, 24*(2), 129-140. doi:10.1007/s11920-022-01324-5

Lanz, M., & Maino, E. (2014). Family Environment Scale. In A. C. Michalos (Ed.), *Encyclopedia of Quality of Life and Well-Being Research* (pp. 2170-2173). Dordrecht: Springer Netherlands.

Loewy, R. L., Bearden, C. E., Johnson, J. K., Raine, A., & Cannon, T. D. (2005). The prodromal questionnaire (PQ): Preliminary validation of a self-report screening measure for prodromal and psychotic syndromes. *Schizophrenia Research, 79*(1), 117-125. doi:<https://doi.org/10.1016/j.schres.2005.03.007>

Mallorquí-Bagué, N., Bulbena, A., Pailhez, G., Garfinkel, S. N., & Critchley, H. D. (2016). Mind-Body Interactions in Anxiety and Somatic Symptoms. *Harv Rev Psychiatry, 24*(1), 53-60. doi:10.1097/hrp.0000000000000085

Moos, R. H., & Moos, B. S. (1976). A Typology of Family Social Environments. *Family Process, 15*(4), 357-371. doi:<https://doi.org/10.1111/j.1545-5300.1976.00357.x>

Morales-Muñoz, I., Palmer, E. R., Marwaha, S., Mallikarjun, P. K., & Upthegrove, R. (2022). Persistent Childhood and Adolescent Anxiety and Risk for Psychosis: A Longitudinal Birth Cohort Study. *Biological Psychiatry, 92*(4), 275-282. doi:<https://doi.org/10.1016/j.biopsych.2021.12.003>

Mujahid, M. S., Diez Roux, A. V., Morenoff, J. D., & Raghunathan, T. (2007). Assessing the Measurement Properties of Neighborhood Scales: From Psychometrics to Ecometrics. *American Journal of Epidemiology, 165*(8), 858-867. doi:10.1093/aje/kwm040

Nishiyama, T., Sumi, S., Watanabe, H., Suzuki, F., Kuru, Y., Shiino, T., . . . Hirai, K. (2020). The Kiddie Schedule for Affective Disorders and Schizophrenia Present and Lifetime Version (K-SADS-PL) for DSM-5: A validation for neurodevelopmental disorders in Japanese outpatients. *Comprehensive Psychiatry, 96*, 152148. doi:<https://doi.org/10.1016/j.comppsych.2019.152148>

Phinney, J. S., & Ong, A. D. (2007). Conceptualization and measurement of ethnic identity: Current status and future directions. *Journal of Counseling Psychology, 54*(3), 271-281. doi:10.1037/0022-0167.54.3.271

Rapee, R. M. (2012). Family factors in the development and management of anxiety disorders. *Clinical child and family psychology review, 15*(1), 69-80. doi:10.1007/s10567-011-0106-3

Rienks, K., Salemink, E., Laas Sigurðardóttir, L. B., Melendez-Torres, G. J., Staaks, J. P. C., & Leijten, P. (2025). Supporting parents to reduce children's anxiety: A meta-analysis of interventions and their theoretical components. *Behaviour Research and Therapy, 185*, 104692. doi:<https://doi.org/10.1016/j.brat.2025.104692>

Robinson, S., Sobell, M., & Leo, G. (2012). Reliability of the Timeline Followback for cocaine, cannabis, and cigarette use. *Psychology of Addictive Behaviors, in press*.

Rowe, R., Costello, E. J., Angold, A., Copeland, W. E., & Maughan, B. (2010). Developmental pathways in oppositional defiant disorder and conduct disorder. *Journal of abnormal psychology, 119*(4), 726-738. doi:10.1037/a0020798

Sandstrom, A., Uher, R., & Pavlova, B. (2020). Prospective Association between Childhood Behavioral Inhibition and Anxiety: a Meta-Analysis. *Journal of abnormal child psychology, 48*(1), 57-66. doi:10.1007/s10802-019-00588-5

Schaefer, E. S. (1965). A configurational analysis of children's reports of parent behavior. *Journal of consulting psychology, 29*(6), 552-557. doi:10.1037/h0022702

Seok, B. J., Jeon, S., Lee, J., Cho, S. J., Lee, Y. J., & Kim, S. J. (2020). Effects Of Early Trauma and Recent Stressors on Depression, Anxiety, and Anger. *Front Psychiatry, 11*, 744. doi:10.3389/fpsyt.2020.00744

Sharif, I., Wills, T. A., & Sargent, J. D. (2010). Effect of Visual Media Use on School Performance: A Prospective Study. *Journal of Adolescent Health, 46*(1), 52-61. doi:<https://doi.org/10.1016/j.jadohealth.2009.05.012>

Sharif, I., Wills, T. A., & Sargent, J. D. (2010). Effect of visual media use on school performance: a prospective study. *J Adolesc Health, 46*(1), 52-61. doi:10.1016/j.jadohealth.2009.05.012

Singh, G. K. (2003). Area deprivation and widening inequalities in US mortality, 1969-1998. *American Journal of Public Health, 93*(7), 1137-1143. doi:10.2105/ajph.93.7.1137

Skirbekk, B., Hansen, B. H., Oerbeck, B., & Kristensen, H. (2011). The Relationship Between Sluggish Cognitive Tempo, Subtypes of Attention-Deficit/Hyperactivity Disorder, and Anxiety Disorders. *Journal of Abnormal Child Psychology, 39*(4), 513-525. doi:10.1007/s10802-011-9488-4

Smith T.W., M. P. (1972–2012). NORC at the University of Chicago; Chicago: 2015. *General Social Surveys*, Cumulative Codebook.

Stover, P. J., Harlan, W. R., Hammond, J. A., Hendershot, T., & Hamilton, C. M. (2010). PhenX: a toolkit for interdisciplinary genetics research. *Curr Opin Lipidol, 21*(2), 136-140. doi:10.1097/MOL.0b013e3283377395

Vane, R., Habhab, Z., Corona, F., & Mednick, S. (2021). 183 Influence of Feelings of Neighborhood Safety on Anxiety and Sleep. *Sleep, 44*(Supplement_2), A74-A74. doi:10.1093/sleep/zsab072.182

Whiteside, S. P., & Lynam, D. R. (2001). The Five Factor Model and impulsivity: using a structural model of personality to understand impulsivity. *Personality and Individual Differences, 30*(4), 669-689. doi:<https://doi.org/10.1016/S0191-8869(00)00064-7>

Yan, W., Wang, Y., Yuan, Y., Farid, M., Zhang, P., & Peng, K. (2024). Timing Matters: A Longitudinal Study Examining the Effects of Physical Activity Intensity and Timing on Adolescents' Mental Health Outcomes. *J Youth Adolesc, 53*(10), 2320-2331. doi:10.1007/s10964-024-02011-9

Yang, Y., Zhou, Y., Zhang, H., Kou, H., Zhao, J., Tian, J., & Guo, C. (2024). Social anxiety undermines prosocial behaviors when required effort. *International Journal of Clinical and Health Psychology, 24*(4), 100533. doi:<https://doi.org/10.1016/j.ijchp.2024.100533>

Zimmerman, M., Thompson, J. S., Diehl, J. M., Balling, C., & Kiefer, R. (2020). Is the DSM-5 Anxious Distress Specifier Interview a valid measure of anxiety in patients with generalized anxiety disorder: A comparison to the Hamilton Anxiety Scale. *Psychiatry Research, 286*, 112859. doi:<https://doi.org/10.1016/j.psychres.2020.112859>

Zucker, R. A., Gonzalez, R., Feldstein Ewing, S. W., Paulus, M. P., Arroyo, J., Fuligni, A., . . . Wills, T. (2018). Assessment of culture and environment in the Adolescent Brain and Cognitive Development Study: Rationale, description of measures, and early data. *Developmental Cognitive Neuroscience, 32*, 107-120. doi:10.1016/j.dcn.2018.03.004
